# Modified Electrospun Polymeric Nanofibers and Their Nanocomposites as Nanoadsorbents for Toxic Dye Removal from Contaminated Waters: A Review

**DOI:** 10.3390/polym13010020

**Published:** 2020-12-23

**Authors:** Badr M. Thamer, Ali Aldalbahi, Meera Moydeen A, Mostafizur Rahaman, Mohamed H. El-Newehy

**Affiliations:** Department of Chemistry, College of Science, King Saud University, Riyadh 11451, Saudi Arabia; bthamer@ksu.edu.sa (B.M.T.); malhameed@ksu.edu.sa (M.M.A.); mrahaman@ksu.edu.sa (M.R.); melnewehy@ksu.edu.sa (M.H.E.-N.)

**Keywords:** nanofibers, polymers, functionalization, nanocomposites, adsorption, dyes, wastewater

## Abstract

Electrospun polymer nanofibers (EPNFs) as one-dimensional nanostructures are characterized by a high surface area-to-volume ratio, high porosity, large number of adsorption sites and high adsorption capacity. These properties nominate them to be used as an effective adsorbent for the removal of water pollutants such as heavy metals, dyes and other pollutants. Organic dyes are considered one of the most hazardous water pollutants due to their toxic effects even at very low concentrations. To overcome this problem, the adsorption technique has proven its high effectiveness towards the removal of such pollutants from aqueous systems. The use of the adsorption technique depends mainly on the properties, efficacy, cost and reusability of the adsorbent. So, the use of EPNFs as adsorbents for dye removal has received increasing attention due to their unique properties, adsorption efficiency and reusability. Moreover, the adsorption efficiency and stability of EPNFs in aqueous media can be improved via their surface modification. This review provides a relevant literature survey over the last two decades on the fabrication and surface modification of EPNFs by an electrospinning technique and their use of adsorbents for the removal of various toxic dyes from contaminated water. Factors affecting the adsorption capacity of EPNFs, the best adsorption conditions and adsorption mechanism of dyes onto the surface of various types of modified EPNFs are also discussed. Finally, the adsorption capacity, isotherm and kinetic models for describing the adsorption of dyes using modified and composite EPNFs are discussed.

## 1. Introduction

Water pollution is one of the most serious problems that has resulted from industrial development and rapid population growth. This problem has exacerbated over time and has become a global problem due to the increasing the amount of wastewater and its discharge into water systems. Dyeing wastewater is one of the riskiest wastewaters that causes water pollution because of its ability to change the color and the properties of water even in the presence of very low concentrations. Dyeing wastewater usually result from many industries such as textile, cosmetic, tannery, photographs, food and plastic industries. Annually, the estimated production of dyes is around 1.6 million tons and 10–15% of dyes are discharged as wastewater to the environment [1]. The main source of colored wastewater is from the textile industry, as it depends on water for most of its operations and the amount is estimated at 200 million liters annually [2]. The releasing of colored wastewater to aquatic systems causes many problems for living organisms due to the dye molecules, heavy metals and aromatic compounds contained in the water, as well as their stability and their ability to reduce sunlight transmission. To avoid these problems, dyeing wastewater must be treated before being released to the environment. In recent years, researchers have made great efforts in testing and developing various techniques to remove dyes from wastewater such as coagulation [3], chemical oxidation [4], biodegradation [5], ultrasonic degradation [6], photodegradation [7], membrane separation [8] and adsorption [9,10,11]. As a result of the high stability of dyes and the possibility of degradation into more toxic molecules, physical treatments such as adsorption are preferred. Since the adsorption process depends mainly on the properties and cost of the adsorbent, most efforts have been focused on producing highly efficient and low-cost adsorbents. With the advent of nanotechnology, various nanomaterials have been used as promising and effective adsorbents and as an alternative for conventional adsorbents. Among these nanomaterials, electrospun polymer nanofibers (EPNFs) have received much attention from researchers in the last two decades, whether in the field of water treatment or other fields [12]. More than 100 natural and synthetic polymers can be converted into nanofibers by dissolving them in a suitable solvent followed by spinning using electrospinning technique to produce EPNFs [13]. EPNFs as one-dimensional nanostructures have unique properties such as a large surface area, high surface-to-volume ratio, high porosity, large number of adsorption sites and high adsorption capacity. These properties allow the use of EPNFs as effective adsorbents for various pollutants such as heavy metals, dyes and others pollutants. To date, there are few reviews on EPNFs and their applications in water treatment. Most of them focus on discussing fabrication and applications of EPNFs in the field of water treatment in general, whether their applications as adsorbents and filtration for various pollutants, as sensors to detect pollutants or as nanocomposites for the degradation of pollutants [14,15,16,17,18,19]. Recently, Pereao et al. published in the *Journal of Polymers and the Environment* and discussed the applications of EPNFs in water treatment and the discussion was limited only to removing heavy metals [20]. Due to the unique properties of EPNFs, a systematic survey and analysis of modifying methods of EPNFs and the role of this in improving their ability to remove various toxic dyes from aqueous systems are useful for researchers to identify suitable methods of improving surface properties of EPNFs and their adsorption capacity and the main problems facing their application as adsorbents for various toxic dyes. Therefore, in this review, we highlight the progress in the preparation and surface functionalization of EPNFs as well as their applications in the removal of toxic dyes from wastewater. This review article will focus mainly on the fabrication of EPNFs, factors affecting on their adsorption capacity, their surface modification, crosslinking, as well as their nanocomposites with carbon nanomaterials, clay, silica, metal oxides, metal-organic frameworks (MOFs) and bacteria into EPNFs mats and their applications as adsorbents for dyes and as an adsorption mechanism.

## 2. Fabrication of EPNFs

**Figure 1:**
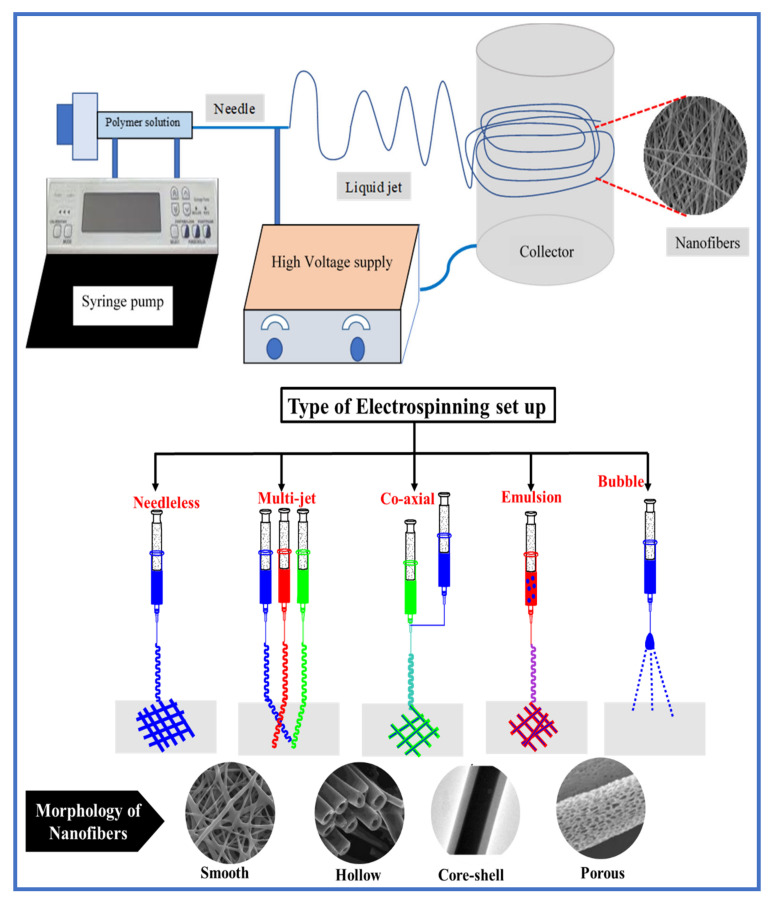
Schematic of types of electrospinning setup and morphology of nanofibers.

Electrospinning is one of the most popular techniques used in the production of polymer nanofibers within the last two decades. This is because of its diversity and simplicity, and its ability to produce nanofibers in various forms with diameters varying from less than one nanometer to several micrometers [21,22]. The setup of this technique is simple and it consists of four main parts: high-voltage power supply, syringe pump, spinneret and collector (Figure 1). For the fabrication of EPNFs, polymer is dissolved in a suitable solvent to obtain a polymer solution with a specific viscosity, then it is spun by applying high voltage with either alternating current (AC) or direct current (DC). Initially, the viscous solution of polymer is pushed and controlled by the syringe pump and ejected from the spinneret as droplets that take a spherical shape due to surface tension. By applying high voltage, the spherical drops on the spinneret are charged with similar charges, which creates electrostatic repulsive forces between them that work to overcome the forces of surface tension and thus turn into a Taylor cone [23]. After formation of the Taylor cone, the jet initially stretches in a straight line and then undergoes strong whisking movements during transferring towards the collector due to the instability of the curvature. When the jet turns to thin fibers as it moves towards the collector, rapid evaporation of the solvent occurs and therefore the nanofibers are deposited onto the collector surface. The morphology and diameters of the formed nanofibers depend on the processing conditions of electrospinning (e.g., applied voltage (V), distance between the spinneret and the collector (TCD), flow rate (F.R), geometry of collector and diameter and geometry of spinneret) polymer solution (e.g., concentration, molecular weight, conductivity, viscosity and surface tension) and ambient conditions (e.g., temperature, humidity and air speed). For example, applied voltage is one of the factors that increase the spinnability of polymer solution and it must be sufficient, whereas if the lower voltage is applied it may not be enough to overcome the surface tension of the polymeric solution droplet. The insufficient or high voltage causes the droplets from the tip of the needle, which produces the beaded nanofibers. Flow rate of the solution definitely affects the fiber formation. The higher flow rates lead to the formation of beaded fibers due to the incomplete dryness of the fiber jet from the needle to the collector [24]. So, the required flow rate can be fixed as much as minimum value to produce a beadles and uniform nanofiber [25]. Theron et al. studied the relationship between the flow rate and applied voltage using different polymeric systems like polyvinyl alcohol (PVA), polycaprolactone (PCL), polyethylene oxide (PEO) and polyurethane (PU). The increase in the flow rate and voltage may decrease the charge density, which causes the merging of nanofibers before depositing on the collector [26]. The conductivity of the solution plays an important role in the fiber formation. The low conductive or the polymeric solutions with no conductivity, will result in the surface of droplet having no charges to form a Taylor cone [27]. The conductivity of polymeric solution needs to increase to a certain level, which may increase the charge density on the surface of the droplet leading to the formation of a Taylor cone. Increasing conductivity beyond the limit may also affect the fiber morphology. The bead-free nanofiber morphology can also be affected by the concentration of the polymeric solution and, at very low concentrations, the applied electric field and surface tension breaks the polymeric chains into small fragments before reaching the collector which may cause the formation of a beaded nanofiber [28]. The distance between the needle and collector is an important parameter that affects the quality of formed nanofibers. A minimum distance between the needle and collector may help to complete the evaporation of solvent before reaching the collector that cause the formation of bead free nanofiber. These distances can be adjusted according to the applied voltage [29]. In addition to that, the atmospheric condition like temperature and humidity may also affect the morphology of nanofiber mats [30]. By optimizing these parameters, the morphology and diameters of the nanofibers can be obtained according to the desired application of the prepared nanofibers [31,32,33]. Table 1 summarizes the best electrospinning conditions of some common polymers. The morphology of the formed nanofibers can also be varied according to the type of technique used, such as needleless electrospinning [34], multi-jet electrospinning [35], bubble electrospinning [36], electro blowing [37], co-axial electrospinning [38], emulsion electrospinning [39] as shown in Figure 1. A single layered nanofiber mat is typically fabricated by the simple electrospinning method [39]. Core shell structured nanofibers are prepared by typical coaxial electrospinning method or simple emulsion electrospinning method [40]. In the coaxial method, two needles are coaxially placed together for two different polymeric solutions. The core material, which was pumped through the inner needle, whereas the shell material was pumped from the outer needle of the coaxial spinneret [41]. The main parameter for this method is that the shell material is likely to be in the electrospinnable polymer solution, whereas the core material is likely to be a drug solution or other non-spinnable material. Multi-layered nanofibers can be prepared by triaxial electrospinning set up, which consisted of three needles connected to the spinneret device core, intermediate and sheath solutions. In this method, core and intermediate solutions should be immiscible, whereas outer sheath and intermediate solutions can be miscible to each other [42]. Core-shell and multilayered nanofibers are mainly used for biomedical application due to the high loading capacity and easiness of poor soluble drug loading into the polymeric shell [43].

## 3. Modification of Polymer Nanofibers

**Figure 2:**
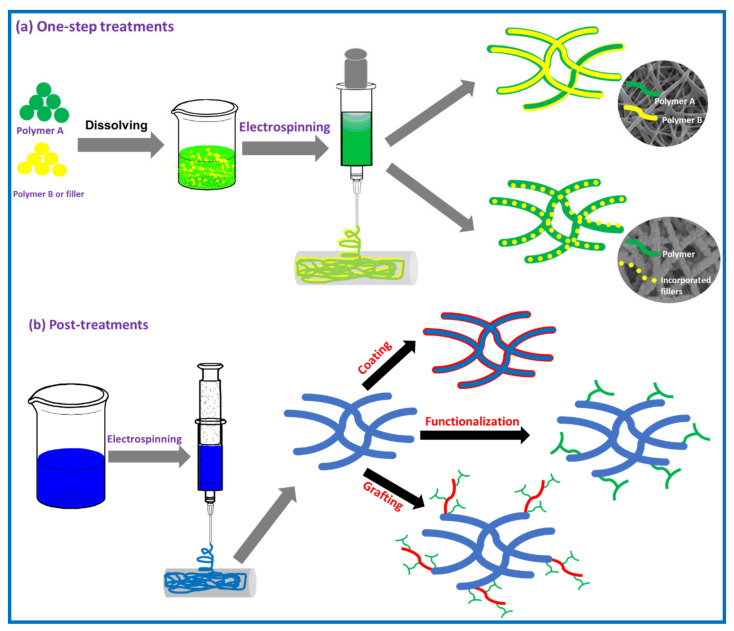
Surface modification of electrospun polymer nanofibers.

Although the advantages of polymer nanofibers include their high surface area-to-volume ratios and high porosity, some pristine polymers still have limitations in the adsorption process [18]. Some have a lack of sufficient adsorption capacity for the removal of pollutants (e.g., polyacrylonitrile (PAN) and nylon), some of them are unstable in aqueous solutions (e.g., PVA, polyacrylic acid (PAA) and polyvinyl pyrrolidone (PVP)) and some of them have low mechanical properties such as chitosan. To overcome these problems, researchers have made great efforts to improve the properties of nanofibers via surface modification. The surface modification aims to enhance the stability of nanofibers in aqueous solutions, improve their hydrophilicity and wettability properties, increase their adsorption sites on the surface, and improving their mechanical properties [55,56]. The surface modification of nanofibers can be carried out by two methods; one-step treatments that can be carried out during electrospinning (nanocomposites and blends) and post-treatments that can be carried out after electrospinning (e.g., plasma, wet-chemistry, grafting and coating) as shown in Figure 2.

### 3.1. One-Step Modification

#### 3.1.1. Blending with Other Polymers

The properties of pristine polymer nanofibers can be enhanced via blending of polymer with another polymer in certain ratios [57]. These properties depend on the ratio, surface energy and molecular weight of the added polymer as well as solvent [56]. The blending method was used to improve the surface properties of several hydrophobic polymers such PVDF [58] polystyrene [59], PET [60], PCL [61], PES [62]. Hydrophobic nanofibers that were used for the removal of ionic pollutants from aqueous solutions are often modified by adding hydrophilic homopolymer or amphiphilic copolymers before the electrospinning process. For example, Ghani et al. modified the surface of polyamide-6 nanofibers by blending with different ratios of chitosan [63]. They found that the hydrophilicity of polyamide-6 nanofibers increased by increasing the ratio of chitosan, and subsequently their adsorption capacity towards the removal of anionic dyes increased. In another study, Xu et al. fabricated blend of PES/P(MMA-co-AA) nanofibers by one-step electrospinning process [62]. They found that the hydrophilicity increased with increasing the ratio of P(MMA-co-AA) as well as their adsorption capacity towards the removal of cationic (MB) dye.

#### 3.1.2. Incorporating Nanomaterials

The incorporation of nanomaterials into the polymer nanofibers by dispersing them into the polymer solution and then electrospun is one of the appropriate methods for modifying the surface properties and mechanical properties of polymer nanofibers as well as improving their adsorption capacity. Incorporated nanomaterials must have a good dispersion property in the polymer solution and have the ability to segregate to the surface of nanofibers so that the modification process is effective. Carbon nanomaterials (e.g., MWCNTs-COOH and GO), metal oxide NPs, nano-clay, MOFs and bacteria are the most incorporated materials that used for modifying the surface of polymer nanofibers and applied for the removal of various pollutants.

### 3.2. Post-Treatment Methods

#### 3.2.1. Wet Chemistry

A wet modification is an effective method for the functionalization of the surface of EPNFs, which can be through a chemical reaction between the surface of the nanofibers and functionalized agent in the solution. The surface properties of the modified electrospun polymer nanofibers depend on the nature of the functional agents. This method depends on the presence of functional groups on the surface of EPNFs that are susceptible to reacting with various agents. Polar functional groups on EPNFs can be created through the hydrolysis and aminolysis processes. For example, hydrolysis of polyester, PCL, PLGA and PBGL by alkali solution are the most wet-chemistry reaction for changing the surface properties of nanofibers [64,65,66]. Another example of wet chemistry modification, the hydrophilicity of PAN nanofibers was enhanced by converting the nitrile groups of PAN into more polar groups such as carboxylic, amino and amidoxime groups [67,68].

#### 3.2.2. Surface Grafting

Grafting method is an effective way to improve the surface properties of EPNFs such as hydrophilicity and their ability to adsorb ionic pollutants by introducing multiple functional groups [69]. The grafting process is carried out through two approaches via grafting-onto or grafting-from. The grafting-from requires the creation of initiators onto the surface of the nanofibers, which can be created by plasma, wet-chemistry and UV radiation treatment or by incorporating initiator int onto the surface of nanofibers during electrospinning [56]. For example with grafting-from, the PVDF nanofibers were modified with PMAA by plasma-induced graft copolymerization results in increasing its hydrophilicity as well as water flux performance [70]. It was found that the hydrophilicity increased after grafting process as well as water flux performance. Electrospun PVC nanofibers were modified with different hydrophilic polymers (e.g., PVP, PAA, PAM and PDMAEMA) by graft polymerization via pre-treatment with AIBN in acetone to produce free radicals on the surface followed by immersion of PVC nanofibers into monomer solution with heating under nitrogen [71]. Contact angle results revealed that the free radical grafting by hydrophilic monomer converted acquired the surface of PVC nanofibers hydrophilic character. In contrast, grafting-onto method requires pre-polymer preparation and then subsequently reacts with the surface of electrospun nanofibers. For example, polydopamine was grafted onto the surface of electrospun PAN nanofibers [72]. Initially, the surface of PAN nanofibers functionalized by DETA followed by grafting with polydopamine in the presence of glutaraldehyde.

#### 3.2.3. Surface Coating

Surface coating is carried out by depositing functional material onto the surface of nanofibers with a layer thickness ranging from a few nanometers to several micrometers. The bonding type between them is physical such as electrostatic interaction, π–π interaction and hydrogen bonding interaction. The surface properties such as hydrophilicity and adsorption capacity will depend on the polarity, layer thickness and the homogeneity of the coating material. The coating technique can be completed by various processes such as immersion nanofiber mats into the material solution, self-assembly, layer-by-layer, electrospraying and polymerization onto the surface of nanofibers. For example, PAN nanofibers were fabricated by electrospinning followed by coating with chitosan through the immersion process and was used for dye removal [73]. The results confirmed that the coating PAN nanofibers with chitosan layer changed their hydrophobic surface to hydrophilic surface, which enhance the adsorption capacity by 28 times. In another study, polylactic acid (PLLA) nanofibers were coated via polymerization of aniline onto their surface. In a similar study, nylon-6 nanofibers were coated with polyaniline by in-situ polymerization onto their surface with average thickness of 65 nm [74]. As another example, the surface of PCL/PEO blend nanofibers was coated by in-situ oxidation process by self-polymerization of polydopamine [75]. The surfaces of polymer nanofibers can be modified by coating them with non-polymeric materials such as metal oxides. The surface of cellulose nanofibers was coated with a few layers of manganese oxide nanosheets by the in-situ method, which revealed high adsorption capacity for the removal of MB [76].

#### 3.2.4. Plasma

**Figure 3:**
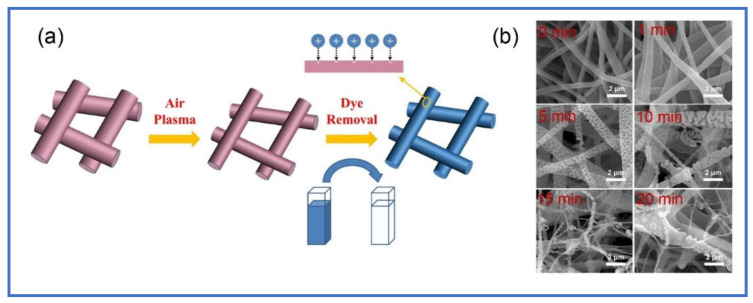
(**a**) plasma-treated polylactic acid (PLLA) electrospun membrane (**b**) effect of duration plasma treatment on morphology of PLLA nanofibers [79]. Copyright © (2020) Elsevier.

Plasma is one of the post-treatment methods used to increase the adsorption sites onto the surface of nanofibers [77]. The plasma treatment depends on using the ionized gases to create polar functional groups (e.g., -OH, -COOH, -OOH, -NH_2_) onto the surface of polymer nanofibers. The introduction of polar functional groups leads to improvement for the hydrophilicity of EPNFs as well as their ability towards the adsorption of ionic materials onto their surface. A number of hydrophobic polymer nanofibers can be treated by plasma such as PCL [78], PLLA [79], PLGA [79] and PVDF [80]. Bai et al. modified the surface of PLLA nanofibres by plasma etching treatment, which lead to changing in the surface of EPNFs from smooth to rough with porous structure and high surface area within 5 min of treatment as shown in Figure 3 [79].

## 4. Factors Affecting the Efficiency of Nanofibers for the Adsorption of Dyes

The adsorption process is a complex process and depends on many factors including the properties of nanofibers, the structure of dyes and the medium of adsorption process.

### 4.1. Effect of Physio-Chemical Properties of Nanofibers

The adsorption capacity of dyes onto the surface of electrospun polymer nanofibers depends on the functional groups, their surface area, their porosity and hydrophilicity/hydrophobicity nature.

#### 4.1.1. Functional Groups

The properties of polymers differ from each other according to the nature of the functional groups. The adsorption efficiency of polymeric nanofibers varies according to the nature of the functional groups onto their surface. The functional groups can be either in the main structure of polymer nanofibers, or created via surface modification methods.

A number of studies have proved that the oxygen-containing functional groups and nitrogen-containing functional groups play a pivotal role in interaction between nanofibers and dyes. For example, the adsorption capacity of PAN nanofibers towards removal of MB and RB was lower than of EDA-PAN nanofibers [81]. This difference in the adsorption capacity is due to the high hydrophilicity and nucleophilicity properties of amino groups compared to the nitrile groups. In a similar study, it was found that converting a nitrile group of PAN nanofibers to an oxime group increased the adsorption capacity from 42.662, 72.46 and 99.30 mg/g to 102.1, 118.3 and 221.2 mg/g for the adsorption of MB, ST, RB, respectively [82]. Chunyan et al. functionalized PES nanofibers by PMETAC and observed the adsorption capacity of modified PES nanofibers for removal of CR dye was higher than of unmodified PES nanofibers [83]. Cheng et al. fabricated and modified cellulose acetate nanofibers and they found that the adsorption capacity of polydopamine-modified nanofibers for the removal of MB was 8.6 times higher than unmodified cellulose acetate nanofibers. This is due to the amine and catechol groups of polydopamine that increase the active adsorption sites onto the surface of cellulose acetate nanofibers.

#### 4.1.2. Surface Area and Porosity

The surface area and porosity of many adsorbents plays a pivotal role in the adsorption process. High surface area and porosity of polymer nanofibers enhances the adsorption performance due to the creation of active adsorption sites. Chen et al. [84] fabricated negatively charged polyethersulfone nanofibers by in-situ cross-linking co-polymerization followed by electrospinning and used as adsorbent for the removal of MB. They found that the adsorption capacity of fabricated nanofibers towards the removal of MB was directly proportional to their surface area and porosity. Shourijeh et al. prepared porous PVDF/PAN nanofibers by electrospinning process through incorporation of different salts [85]. They found that the adsorption capacity of all type of porous PVDF/PAN nanofibers for removal of BB-41 dye was higher than nonporous fibers. Wang et al. [39] fabricated PCL/PEO by electrospinning and modified with polydopamine. They observed that the surface area and porosity of the prepared nanofibers increased by modification leading to enhancement of their adsorption capacity [75]. Guo et al. [50] observed that the surface area and porosity of PCL nanofibers can be increased after surface modification by beta-cyclodextrin leading to increase in their adsorption capacity towards the removal of MB dye [86]. Additionally, one of the factors that has a direct relationship to the surface area of EPNFs and may play a role in their efficiency is the diameter. Li et al. studied diameter’s effect on adsorption capacity of chitosan nanofibers towards the removal of AB-113 dye [87]. They found that the relationship between diameters and adsorption capacity of EPNFs is an inverse relationship, as the adsorption capacity increased from 867 to 1338 mg/g, with a decrease in diameter from 164 ± 28 to 86 ± 18 nm.

#### 4.1.3. Hydrophilicity/Hydrophobicity of Nanofibers

Hydrophilic EPNFs with their polar functional groups have higher adsorption capacity towards the removal of dyes compared to hydrophobic one. Polar functional groups play an important role in wettability of nanofibers surface as well as in reducing the repulsive forces between the surface and the dyes molecules in an aqueous solution. For example, Liu et al. observed that the adsorption capacity of copolyester nanofibers increased from 49.90 to 543.48 mg/g while the contact angle decreased from 135° to 46° upon surface functionalization with carboxymethyl-β-cyclodextrin [88]. In another study, they was found that the functionalized cellulose acetate nanofibers by polydopamine enhanced their hydrophilicity and improve their adsorption capacity for the removal of MB dye [89].

### 4.2. Effect of Dye Nature

Adsorption of dyes onto the surface of polymer nanofibers and their nanocomposites depends on their molecular structure, molecular size and functional groups. Table S1 displays the chemical structure and physical properties of some dyes that were used as models to test the efficiency adsorption of the nanofibers in literature. For instance, Min et al. investigated the adsorption of three types of anionic dyes (e.g., Sunset Yellow FCF, Fast Green FCF, Amaranth) onto the surface of PES/PEI nanofibers [90]. They found that the adsorption capacity of PES/PEI nanofibers was 1000, 454.55 and 344.83 mg/g for the SY FCF (M.W: 452.38g/mol) AM (M.W: 604.47 g/mol) and FG FCF (M.W: 808.85 g/mol), respectively. These results indicated the molecular weight of the dye has a contributory influence on the adsorption capacity. Dogan et al. fabricated P(HPβCD)/P(BA-a) nanofibers and studied their adsorption for MB and MO [91]. They found that the adsorption affinity of MB onto the prepared nanofibers was higher than of MO. This was attributed to the positively charged MB that enhances the electrostatic interaction with P(HPβCD15/P(BA-a) nanofibers. Zhan et al. observed that the adsorption of cationic dye onto the surface of ZIF-8/PAN nanofibers depends on the molecular structure of dye [92]. They concluded that the adsorption capacity of MG (1666.67 mg/g) was much higher than of MB (120.48 mg/g) due to the interaction between aromatic ring of MG and double bonds and a pair of electrons in imidazole moieties. Table S1 summarizes the chemical structure and maximum absorption wavelength (λ_max_, (nm) of the dyes addressed in this review.

### 4.3. Effect of Operating Conditions of Adsorption

The removal of dyes from aqueous solution by adsorption process is highly dependent on operating conditions like pH, adsorbent dosage, initial concentration of dye, contact time and temperature.

#### 4.3.1. pH Effect

The pH is one of the most important factors that affect the adsorption process, due to its effect on the ionization degree of functional groups for both polymer nanofibers and dye. In general, increasing the pH of the adsorption medium enhances the adsorption of cationic dyes due to increasing the electrostatic attraction between the dye and polar groups on the surface of nanofibers and vice versa with anionic dyes. Several studies have examined the effect of pH on the adsorption capacity of polymer nanofibers and their nanocomposites towards dyes removal from an aqueous system. Fan et al. performed the adsorption of cationic dye (MB) and anionic dye (MO) at various pH onto PANF-g-HPEI nanofibers [93]. They found that the maximum adsorption capacity and adsorption rate were happen at pH 3 and pH 10 for MO and MB, respectively. The adsorption dependence on pH confirmed that the electrostatic interaction between the dye molecules and the nanofibers surface is dominant. Mousavi et al. studied the adsorption-desorption of MB onto porous nanofiber aerogels that fabricated by electrospinning of pullulan/PVA/PAA followed by thermal crosslinking [94]. They found that the basic medium (pH 11) was suitable for the adsorption of MB onto fabricated nanofiber aerogel, while the desorption process was achieved in acidic medium (pH 2.6). This was due to the deprotonation of carboxylic and hydroxyl groups in basic medium, which enhances the electrostatic interaction between negatively charged surface of nanofibers and cationic ions of MB.

#### 4.3.2. Nanofibers Dosage

Optimization of adsorbent doses required for complete removal of dye from aqueous solutions is very important to determine the cost-effectiveness of adsorbent. In general, the dye removal percentage increases with increasing the adsorbent dose until certain dose due to the increase of adsorption sites on the adsorbent surface with increasing the amount of adsorbent. In contrast, the adsorption capacity decreases with increasing adsorbent dose due to the mathematical inverse proportional to the adsorbent dosage. Mahmoodi et al. studied the effect of ZIF-8@chitosan/PVA nanofibers dose on the removal of MG dye and they found that the removal percentage increase with increasing the adsorbent dose and the optimum dosage was 0.03 g/L [95]. Almasian et al. also investigated the effect of PAN/Tectomer nanofiber dosage on removal percentage of DR-80 and DR-23 and they observed that the removal percentage was increased with increasing the adsorbent dosage [96].

#### 4.3.3. Contact Time

The contact time factor helps in determining the optimum time required to saturate the active adsorption sites on the surface of the nanofibers and complete removal of dye. The adsorption efficiency and adsorption capacity usually increase by increasing the contact time to reach the equilibrium state, then remains steady state thereafter. Most of the studies that used polymer nanofibers as an adsorbent to remove various types of dyes studied the effect of contact time and determined the time required to reach equilibrium. For example, Soltan et al. studied the effect of contact time on the removal of anionic dyes using nylon-6/poly(propylene imine) dendrimer nanofibers [97]. They found that within the first 2 minutes, more than 90% of the dye was removed. Lu et al. studied the effect of contact time on the adsorption capacity of PAN/MoS_2_ nanofibers at different concentration of RhB dye [98]. They found that the adsorption capacity increased rapidly in the first few minutes and reached equilibrium after 50 min for an initial concentration less than 100 mg/L and 180 min for an initial concentration higher than 250 mg/L. San et al. investigated the required contact time to reach equilibrium state for removal of MB using cellulose acetate/bacteria nanofibers [99]. They found that the highest percentage removal of MB dye was obtained after 48 h. Therefore, the optimum contact time needed to reach the equilibrium state for the adsorption of dyes depends on the physical and chemical properties of the nanofibers as well as the nature of the incorporated materials inside nanofiber mats.

#### 4.3.4. Initial Dye Concentration

The adsorption capacity of the nanofibers largely depends on the initial concentration of the dye in the aqueous solution. In general, the adsorption capacity increases with increasing dye concentration due to the increase in the number of dye molecules in the contact area between the surface of the nanofibers and aqueous solution. The increasing in the contact area enhances the driving force of the transferred mass as well as the number of collisions between dye ions and nanofibers surface. In contrast, the percentage of dye removal decreases with an increase in the initial concentration of the dye due to saturation of the active adsorption sites on the surface of the nanofibers, which leads to an increase in the remaining concentration of the dye in the aqueous solution. Wang et al. studied the adsorption of MB onto sodium alginate nanofibers that was crosslinked by CaCl_2_ and they found that the adsorption capacity increased from 500 to 2357.87 mg/g with increasing the initial concentration of MB from 200 to 1500 mg/L [100]. Almasiana et al. studied the removal of DR-80 and Dr-23 dye by PAMAM-grafted-PAN-DETA nanofibers at different initial concentration of dye [72]. They found that the dye removal percentage decreased with increasing the initial concentration of dyes.

#### 4.3.5. Temperature

The effect of the temperature on the adsorption of dyes onto EPNFs plays a vital role in interpreting the mechanism of adsorption. In general, increasing the adsorption capacity with increasing the temperature indicates that the adsorption process is endothermic and is exothermic with decreasing the temperature. The increase in the adsorption capacity with increasing the temperature is attributed to higher mobility of the dye molecules and strong interaction between the dye ions and the active adsorption sites on the surface of the nanofibers. For example, the adsorption of MB dye by P(NIPAM-co-βCD)/P(NIAPM-co-MAA) [101] and DCA/PDA [89] nanofibers was an endothermic process while it was exothermic by sodium alginate [100], Keratin [102] and gelatin/β-CD [103] nanofibers. Table 2 summarizes the results of some studies that investigated the effect of temperature on the adsorption of different dyes on the surface of functionalized polymer nanofibers.

## 5. Adsorption Mechanism of Dyes onto Nanofiber Mats

**Figure 4:**
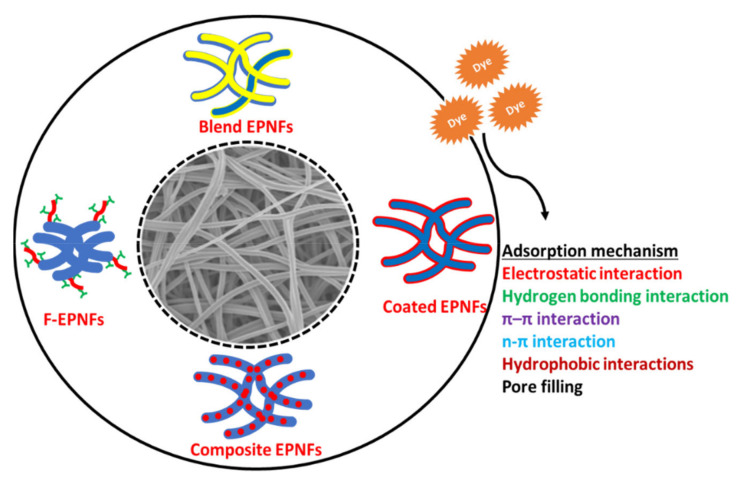
Adsorption mechanism of dyes onto surface EPNFs.

Understanding the adsorption mechanism of dyes onto adsorbent is very essential in order to optimize the adsorption process and improve the nanofiber’s efficiency towards the removal of dyes. Adsorption of dyes onto the surface of nanofibrous materials depends on the conditions of the solution (e.g., pH, temperature), the nature of the nanofibers (e.g., porosity, surface area, functional groups, morphology) and the nature of the dye (e.g., cationic form, anionic form, molecular size). Due to the multiplicity of factors affecting the adsorption process of dyes onto the surface of the nanofibers, the adsorption mechanism may not be explicit. Therefore, it is necessary to conduct isotherm, kinetic, thermodynamic and spectroscopic studies as well as studying the effect of pH to obtain a clear and in-depth view of the adsorption mechanism. As reported from different studies [108,109,110,111,112], the removal of dyes from aqueous solutions using pristine, blend functionalized polymer nanofibers likely occurs via hydrogen bonding, van der Waals forces, π–π stacking, hydrophobic interactions and electrostatic interactions as well as pore filling. Each interaction depends on the nature of the functional groups on the surface of nanofibers, their morphology and the nature of the additive to the nanofibers. Due to the multifunctional groups in polymer nanofibers and their nanocomposites, the adsorption mechanism of dyes can be explained by a combination of these interactions that operate simultaneously with varying degrees. For example, Qureshi et al. studied the adsorption mechanism of Acid Blue 117 dye onto the surface of nylon-6 nanofibers by FTIR and XPS techniques. They concluded that the adsorption of dye achieved through combination of interactions such as hydrophobic, electrostatic and hydrogen bond interactions [113]. Al-Marjeh et al. investigated the adsorption mechanism of MO onto the surface of pTSA-PANI/PLLA by pH and ionic strength effect [114]. They found that the adsorption process depends mainly on the electrostatic interaction between the negative charge of MO and the positive charge of emeraldine salt state of polyaniline on the PLLA nanofibers surface. Chaúque et al. functionalized PAN nanofibers by EDTA and EDA, and studied adsorption of anionic dyes such as MO and RR dye [105]. They found that the adsorption capacity of MO and RR dye onto the surface of EDTA-EDA-PAN nanofibers was higher than pristine PAN nanofibers and stable at various pH values. This result attributes that the adsorption mechanism depends on non-electrostatic interactions such as hydrogen bonding, hydrophobic interactions, and van der Waals forces, and to a lesser degree on the electrostatic interaction. Chen et al. studied the adsorption performance of gelatin/β-cyclodextrin nanofibers towards the removal of MB dye [103]. They concluded that the adsorption mechanism in alkaline medium depends on the electrostatic interaction between carboxylic groups of gelatin and positive charge of MB as well as host-guest interaction between β-CD and MB. Figure 4 summarizes adsorption mechanisms of dyes onto EPNFs and their composites.

## 6. Types of EPNFs as Adsorbent for the Removal of Dyes

Polymers are characterized by high molecular weights and are divided according to the source into two main parts: natural and synthetic polymers. Some polymers have been used as adsorbents for many pollutants, including dyes, especially those containing effective functional groups and insoluble in water. The efficiency of polymers in dye removal depends on the effective group nature, morphology, surface area and porosity. Converting polymers into nanofibers is one of the most effective ways to improve their efficiency in removing dyes due to the unique properties of nanofibers such as high-water permeability, high surface area and porosity.

### 6.1. Homopolymers-Based-EPNFs as Adsorbent for Dyes Removal

Polyamide nanofibers are one of the common homopolymers that have been used as an effective adsorbent for the removal of dyes as it is inexpensive, insoluble in water, keeps its morphology during the adsorption process and is reusable [115]. For example, Qureshi et al. fabricated nylon-6 nanofibers by an electrospinning technique and used them as an efficient and selective adsorbent for the removal of anionic dyes [113]. The obtained results indicated that the removal efficiency of nylon-6 increased from approximately 20% to 90% upon converting it to nanofibers. The sulfonated polysulfone (SPES) is also considered as a synthetic polymer, is spinnable and contains the active sulfonate and sulfonic groups [116]. Yin et al. prepared ultrafine nanofibers based on SPES and applied them for the adsorption of dyes and heavy metals [117]. Recently, researchers have been focused on the use of natural and electrospinnable polymers due to their abundantly inexpensive and non-toxic properties. Li et al. have successfully fabricated chitosan nanofibers by electrospinning and used as adsorbent for the removal of acid blue-113 from an aqueous solution [87]. They found that the diameter of chitosan nanofibers has a significant effect on the removal efficiency and the adsorption capacity increased from 412 to 1377 mg/g upon converting the chitosan fibers from the micro to nanoscale. Although pure chitosan has a high ability to adsorb the dye because it contains amino and hydroxyl groups, its spinnability is weak. Therefore, in the aforementioned study, Triton-100 as surfactant was added to chitosan solution facilitate the electrospinning process. The Triton-100 is not subject to evaporation during the spinning or drying process and is soluble in water, so it can cause secondary water pollution when chitosan is used to treat water. Another example of biopolymer nanofibers that have been used as an effective bioadsorbent for dyes removal is zein but its ability to remove dyes remains low compared to chitosan nanofibers [118]. In general, the adsorption capacity of pure spinnable polymers for removal of various dyes was low compared to other materials, as Table 5 shows the adsorption capacity of PLLA [79], Keratin [102], Nylone-6 [113], polyamide 6 [115], SPES [117], Zein [118] and PMAA-co-PMMA [119]. 

### 6.2. EPNFs Blends as Adsorbent for Dyes Removal

**Figure 5:**
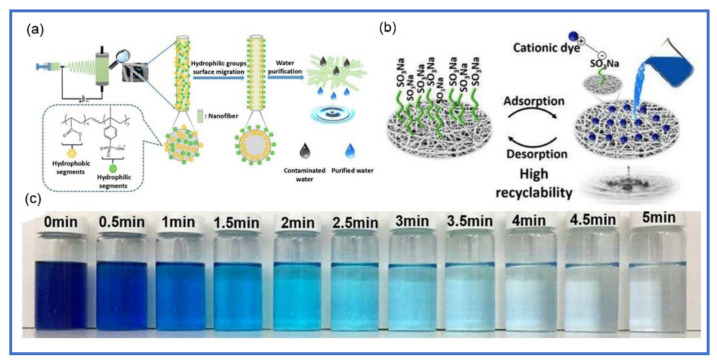
(**a**) Fabrication of blend PES/P(MMA-SSNa) nanofibers (**b**) migration of sulfonate groups into the surface of nanofibers and their role in interaction with MB (**c**) Photography of cationic dye (MB) color change with time after immersing nanofibers [128]. Copyright © (2020) Royal Society of Chemistry.

Due to the low adsorption capacity, low mechanical properties and hydrophobic properties of homopolymers that are spinnable and insoluble in water, the polymer blends have been used as substituent and efficient adsorbents for the removal of dyes from an aqueous system. The blend of a polymer containing functional groups with hydrophobic polymer in different ratio plays an important role in improving its hydrophilicity and increasing the adsorption active sites as well as decreasing the cost [120]. One of the spinnable and hydrophobic polymers that have good mechanical properties is polyacrylonitrile (PAN) [121,122,123]. The adsorption capacity of PAN towards the adsorption of dyes is very low due to its hydrophobic character and lack of effective functional groups. Therefore, many studies have proven that blending of other polymers with PAN nanofibers has improved its ability to remove various dyes. For instance, Hou et al. fabricated a blend of PAN/polyamidoamine (PAMAM) with a certain ratio, which led to increasing its surface area and enhanced its adsorption capacity towards the removal of methyl orange dye [124]. Chitosan and its blends have also shown good adsorption efficiency due to the presence of amine groups in their backbone [125]. Recently, several studies have used blends of chitosan as effective adsorbents for dye removal. Dotto et al. fabricated a blend of chitosan/polyamide (CS/PA) nanofibers by electrospinning and used it as an effective adsorbent for the removal of Ponceau 4R (P-4R) and Reactive Black 5 (RB-5) [126]. They found that the monolayer adsorption capacity of CS/PA nanofibers was 502.4g/g for P4R and 456.9 mg/g at pH 1. Furthermore, they observed that CS/PA nanofibers can be reused four times without decline in its adsorption capacity. Lou et al. demonstrated the ability of chitosan on enhancing the adsorption capacity of PAN towards the removal of Acid Blue 113 from an aqueous solution [73]. They found that the adsorption capacity of PAN nanofibers towards the removal of AB-113 after being coated with chitosan increased from 48.6 mg/g to 1368 mg/g and reached to equilibrium after 2 h. This was attributed to the enhancement of the hydrophilic property of nanofibers after coating with chitosan as well as presence of amino groups. The blend of polymers in different proportions can improve its adsorption capacity and overcome non-electrospinnability of some polymers as well as enhancing their mechanical properties. For example, polyethylenimine is non-spinnable due to the ease of cross-linked and the difficulty to dissolve in a suitable solvent. Ma et al. prepared polyethylenimine nanofibers by electrospinning through mixing it with poly(vinylidene fluoride) [127]. They suggested a ratio of PVDF should exceed 50 wt.% in order to prepare fine nanofibers with less spindles. The synthesized PEI/PVDF nanofibers was used for the removal of MO dye, which exhibited good adsorption capacity (633 mg/g) and can be reused more than 10 times. Xu et al. fabricated PES nanofibers and enhanced its adsorption capacity by blending with amphiphilic copolymer like methyl methacrylate and sodium styrene sulfonate (P(MMA-SSNa) [128]. They found that negatively charged sulfonated groups migrated onto the surface of nanofibers and enhanced their adsorption rate and adsorption capacity towards the removal of MB as shown in Figure 5. Based on the data shown in Table 3, it was found that the adsorption capacity of polymer nanofibers increases when mixed with one or more polymers, due to the creation of new functional groups which increase the adsorption sites on the surface of the nanofibers. Moreover, the best isotherm and kinetic model to describe the adsorption process of cationic and anionic dyes on the surface of homo and blend polymer were Langmuir and PSO models. These results indicate that the surface of the EPNFs was homogeneous and that the amount of dyes adsorbed on their surface was in the form of a single layer.

### 6.3. Crosslinked EPNFs as Adsorbent for Dyes Removal

**Figure 6:**
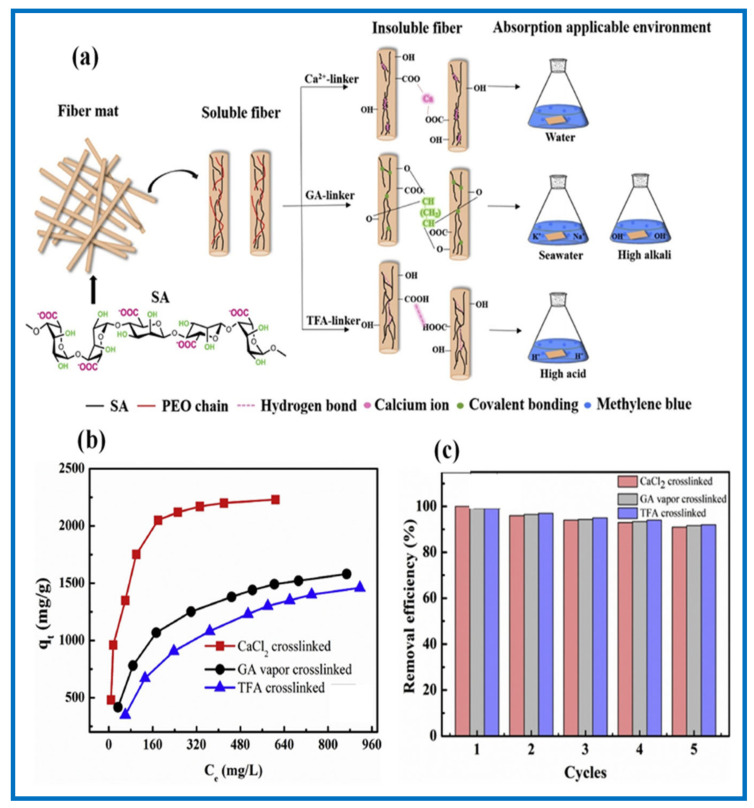
(**a**) Fabrication of sodium alginate /PEO nanofibers and crosslinking by CaCl_2_, GA vapor and TFA (**b**) Adsorption of MB by prepared crosslinked SA/PEO nanofibers (**c**) Reuse of crosslinked SA/PEO nanofibers for adsorption MB [100]. Copyright © (2020) Elsevier.

Water-soluble polymers are one of the most important polymers that used in many applications and can also be converted some of them into nanofibers due to their high electrospinnability. However, the use of nanofibers derived from water-soluble polymers such as poly (vinyl alcohol) (PVA) have limitations for use in water treatments due to their instability in aqueous systems. However, this problem can be overcome by creating crosslinking points on the polymer chains to avoid their solubility in water [129]. The crosslinking between polymer chains can be achieved by creating a chemical bond or by physical interaction. Crosslinking process of electrospun nanofibers derived from water-soluble polymers is achieved after the electrospinning process. For instance, PVA nanofibers can be crosslinked by physical crosslinking (alcohol treatment or thermally) [130,131] or by chemical agents such as glutaraldehyde [132,133]. So, PVA was used to improve the spinnability of some natural polymers such as chitosan [134], starch [135,136], cellulose [137] and Polycyclodextrin [138,139]. Mei et al. easily fabricated PVA/CS nanofibers by electrospinning followed by crosslinked with glutaraldehyde for the adsorption of Congo red (CR) dye [140]. The maximum adsorption capacity for CR dye was 358 mg/g at pH  =  6, 25 °C, dose of nanofibers 6 g/L of 100 mg/L CR solutions. In another study, Moradi et al. fabricated PVA/starch nanofibers with high surface area (24.72 m2/g) by electrospinning followed by thermal crosslinking thermally crosslinked for the removal of cationic dye (MB) [141]. The adsorption capacity for MB was 400 mg/g on the crosslinked PVA/starch nanofibers and the isotherm and kinetic of the adsorption were described by Langmuir and PSO model, respectively. Wang et al. prepared sodium alginate electrospun nanofibers by electrospinning and the crosslinking processes were conducted by various crosslinking agents (calcium chloride (CaCl_2_), glutaraldehyde (GA) and trifluoroacetic acid (TFA)) and was used as a nanoadsorbent for the removal of MB dye as shown in Figure 6 [100]. They found that the tensile strength, surface area, adsorption capacity and adsorption equilibrium time are dependent on the type of crosslinking agent. The maximum adsorption capacity of sodium alginate electrospun nanofibers that were crosslinked by CaCl_2_ was found to be 2230 mg/g, which was higher than that crosslinked by glutaraldehyde and trifluoroacetic acid and the removal efficiency for all remained at 90% after five regeneration cycles as shown in Figure 6a,b. Song et al. successfully prepared two kinds of electrospun crosslinking PEI/PAN nanofibers; during electrospinning (in situ crosslinking) and after electrospinning (solution crosslinking) [142]. Both crosslinking processes were achieved by epichlorohydrin and the maximum adsorption capacity of PEI/PAN nanofibers (by in situ) and PEI/PAN nanofibers (by solution) for MO dye was 636.94 and 595.24 mg/g, respectively. In another study, in situ crosslinking methods have been used by adding monomer of acrylic acid and sodium styrene sulfonate to the PES solution in the presence of initiator followed by electrospinning process [84]. The PSSNa/PAA@PES nanofibers were used for the adsorption and separation of MB from mixture of MB/MO and MB/AR. It was found that the separation efficiency of PSSNa/PAA@PES for MB from mixture of MB/MO and MB/AR was 97.54% and 98.29%, respectively.

The good selectivity towards the adsorption of MB was attributed to the electrostatic attraction between the negatively charged (−SO_3_−) groups on the surface of PSSNa/PAA@PES and the positively charged MB molecules and vice versa. Dogan et al. synthesized crosslinked polycyclodextrin/polybenzoxazine nanofibers (PolyHPβCD/PolyBA-a) by electrospinning blend of hydroxypropyl-β-cyclodextrin benzoxazine and citric acid followed by thermally cured and used for the removal and separation of MB [91]. It was observed that the incorporation of 15% of citric acid into PolyHPβCD/PolyBA-a nanofibers led to the enhancement of its stability in water and organic solvents. Despite its low adsorption capacity for MB (46 mg/g), it showed excellent selectivity to separate MB from a mixture of MB/MO. Table 4 displays the data on the adsorption of various dyes by crosslinked EPNFs. Zhu et al. fabricated crosslinked PVA/PEI nanofibers followed by a coating by dopamine through an in-situ polymerization process [143]. They noticed that the prepared crosslinked nanofibers exhibit good chemical stability in the harsh environments, good mechanical properties and excellent removal efficiency of cationic and anionic dyes. Other crosslinked EPNFs like Gel/Ca-Alg [144], PVA–TETA [145], SS/PVA [146], β-CD/PAA/citric acid [147], SS/β-CD/PVA [148], PVA-CS [149] and CA/P(DMDAAC-AM) [150] also showed good adsorption capacity for the removal of various cationic and anionic dyes from contaminated water. In contrast, crosslinked EPNFs such as P(HPβCD)/PBA-a [91], Gel/β-CD [103], Alg/PEO [151] showed less efficiency towards removing dyes. Based on data in Table 5, the best isotherm and kinetic model to describe the adsorption process of cationic and anionic dyes on the surface of crosslinked EPNFs were Langmuir and PSO model, except one study that found the Freundlich model was the best for describing adsorption of MB onto the surface of PSSNa/PAA@PES [84]. These results indicate that the surface of the functionalized EPNFs was homogeneous and that the amount of dyes adsorbed on their surface was in the form of a single layer.

### 6.4. Functionalized EPNFs as Adsorbent for Dyes Removal

**Figure 7:**
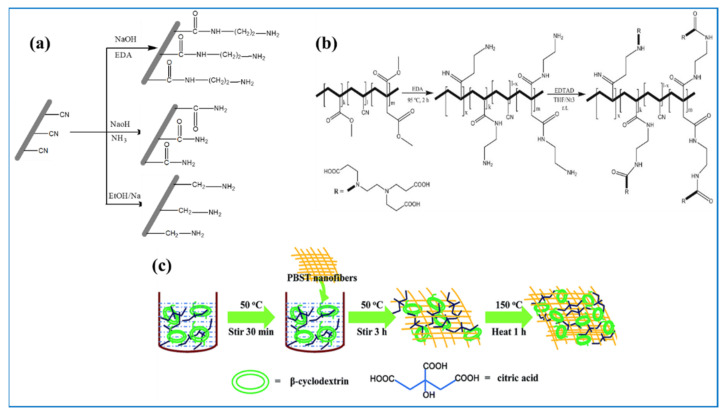
(**a**) surface functionalization of PAN by EDA, NH_3_ and EtOH/Na [67], Copyright © (2020) Elsevier (**b**) surface functionalization of poly(acrylonitrile-methyl acrylate-itaconic acid) nanofibers by EDTA [105], a Copyright © (2020) Elsevier and (**c**) surface functionalization of poly(butylene succinate-co-terephthalate) by cyclodextrin [163], Copyright © (2020) Royal Society of Chemistry.

The surface functionalization of EPNFs plays an important role in enhancing the adsorption capacity for the removal of dyes and other pollutants. Introducing active and hydrophilic functional groups is an effective method to increase the active sites of adsorption onto the surface of nanofibers. The surface functionalization can be carried out by alkaline hydrolysis, chemical grafting method, plasma-induced grafting. PAN is one of the most synthetic polymers that can be easily electrospun into nanofibers and have unique properties such as good stability in aqueous solutions due to its hydrophobic nature as well as its mechanical properties. However, the adsorption ability of PAN nanofibers for dyes removal from aqueous solutions still week due to its hydrophobic nature. Therefore, the surface modification of PAN nanofibers is an effective way to increase its hydrophilicity properties as well as the adsorption sites on the surface with keeping its morphology [154,155,156]. For example, Patel and Hota prepared PAN nanofibers and carboxylate-functionalized by alkaline hydrolysis using sodium hydroxide and sodium bicarbonate [157]. They found that the removal of MG from aqueous solutions increase significantly after functionalization. The adsorption capacity of PAN-COOH was found to be 1038 mg/g at pH 5 and 35 °C. In another study, Haider et al. fabricated and functionalized PAN nanofibers with hydroxylamine hydrochloride to produce oxime grafted PAN and applied as a good adsorbent for the removal of MB, RhB and ST dyes [82]. After oxime grafting, PAN nanofibers keep its morphology and the adsorption capacity for removal of MB, RB and ST increases from 42.66, 72.46 and 99.3 mg/g to 102.1, 118.3 and 221.2 mg/g, respectively. Amine amino-functionalized PAN nanofibers is another route to improve its surface properties such as hydrophilicity and improve its ability to adsorb different dyes. A total of three amino-functionalized PAN nanofibers prepared by Patel and Hota were used as effective adsorbents for the removal of Congo red dye as shown in Figure 7a [67]. It was found that the adsorption capacity depends on the density of amino groups and the number of active sites on the surface of nanofibers. The maximum adsorption capacity of PAN-EDA was 130 mg/g and the adsorption process followed Langmuir and PSO model for describing isotherm and kinetic, respectively. In another study, Chauque et al. functionalized surface of poly (acrylonitrile-methyl acrylate-itaconic acid) with ethylenediamine followed by ethylenediaminetetraacetic acid (EDTA) and was used as adsorbent for the removal of ionic dyes as shown in Figure 7b [105]. They found that the modified nanofibers have good adsorption capacity towards the removal of MO and RR dye and can be used five times without significant decline in their efficiency. Mahmoodi et al. developed novel porous amino-functionalized PAN nanofibers fabricated through incorporation of Na_2_CO_3_ salt into PAN nanofibers by electrospinning followed by leaching and functionalization processes [158]. Amine-functionalization was carried out by triethylenetetriamine and was used for the removal of Direct Blue 78 dye. The porous aminated-PAN nanofibers exhibited superior adsorption capacity (2500 mg/g) towards the removal of Direct Blue 78 dye. Recently, a similar study by Shourijeh et al. prepared porous aminated PAN/PVDF nanofibers by incorporation of NaHCO_3_ salt using electrospinning, followed by leaching and functionalization by diethylenetriamine agent [159]. It was found that the adsorption capacity of the prepared nanofibers was 685.63 mg/g for the removal of DR-23 dye. In another work, Almasian et al. functionalized poly(acrylonitrile-co-vinyl acetate) with polyamidoamine then turned to nanofibers using electrospinning and used as an effective adsorbent for the removal of DR-80 and DR-23 dye [160]. They found that the increase in the ratio of polyamidoamine in the nanofiber mats is accompanied with an increase in fiber diameters and decrease in the surface area. The maximum adsorption capacity increased from 1666.66 to 2000 mg/g with increasing the amount of polyamidoamine from 10 to 20 *w*/*w*%. Chen et al. prepared PVA/CS nanofibers that were functionalized by polyhexamethylene guanidine and used as adsorbent membrane for the removal of Congo red dye [161]. They found that the functionalization results in a slight change in the surface area of the nanofibers with adsorption capacity of 289 mg/g for the removal of CR dye but the reusability was sharply declined after the first cycle. Wang et al. used polydopamine for the functionalization of polycaprolactone/polyethylene oxide nanofibers via in-situ oxidation self-polymerization and was applied for the removal of both cationic and anionic dyes [75]. The synthesized nanofibers exhibited adsorption capacity towards the removal of anionic dye (MO) higher than cationic dye (MB). It is noteworthy that the nanofibers can also be reused more than eight times without a significant change in the adsorption capacity of the MO dye. Another polymer that could be converted into EPNFs and modified its surface by different methods and used as an effective and selective adsorbent for removal of cationic dyes is cyclodextrin [162]. Additionally, in-situ polymerization was used for functionalization surface of EPNFs. For example, poly(butylene succinate-co-terephthalate) functionalized by β-cyclodextrin through the in-situ polymerization and was used for the removal of MB dye as displayed in Figure 7c [163]. It was found that the adsorption capacity of functionalized nanofibers was higher than unmodified nanofibers. Recently, Liu et al. preparation biodegradable aminated copolyesters nanofibers by multi-steps and applied as effective adsorbent for removal of cationic MB dye [88]. They showed that the removal efficiency of MB was 98% after five-time reuse and maximum adsorption capacity was 543 mg/g. According to Table 5, the functionalized EPNFs can be used as effective nanoadsorbents for the removal of various dyes due to their structure containing more than one polymer and different polar functional groups. The best isotherm model to describe the adsorption process of cationic and anionic dyes on the surface of functionalized EPNFs were Langmuir model, except two studies that found the Freundlich model was the best for describing adsorption of MO, RB onto the surface of EDTA-PAN nanofibers [105] and CR dye onto the surface PHMG-OCS-PVA nanofibers [161].

### 6.5. EPNFs Based on Composites Polymers as an Adsorbent for Dye Removal

#### 6.5.1. EPNFs/Clay Nanocomposites

Mineral clays are natural materials, constructed from hydrous aluminum, magnesium and iron silicates and used as low-cost adsorbents for the removal of various pollutants [165]. Clay has been used as a filler to improve the properties of some materials such as polymers and to prepare nanocomposite membranes. Hosseini et al. fabricated novel PVA/CS/montmorillonite nanofiber composites by using electrospinning and utilized as adsorbent for the removal of Basic Blue 41 [166]. They concluded that the incorporation of montmorillonite into PVA/CS led to enhancing their mechanical properties permeability and adsorption capacity. The zeolite was also used as a filler to improve the mechanical properties of some of polymeric nanofibers as well as the adsorption property. Lee et al. fabricated PMMA/zeolite nanofibers as adsorbent for the removal of MO [167]. They found that the maximum adsorption capacity was 95.33 mg/g. Habiba et al. prepared PVA/CS/zeolite using electrospinning for the removal of MO from aqueous solutions [168]. The maximum MO adsorption capacity on PVA/CS/Zeo nanofibers was 153 mg/g at pH 4.0. They also reported that the addition of zeolite to PVA/CS nanofibers led to increase the Young’s Modulus by more than 100%.

#### 6.5.2. EPNFs/Carbon Nanomaterials Nanocomposites

**Figure 8:**
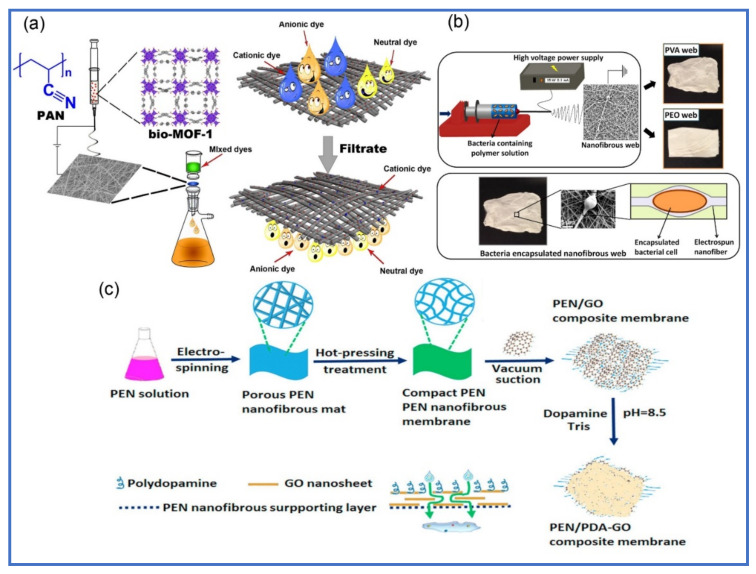
(**a**) Fabrication of bio-MOF/PAN nanofiber composite for removal MB dye [174], Copyright © (2020) Elsevier (**b**) Incorporation bacteria into PVA/PEO nanofibers for removal MB dye [175], Copyright © (2020) Elsevier and (**c**) Fabrication of PAEN/GO-PDA nanofiber composite for removal DB-14 dye [173], Copyright © (2020) Elsevier.

Despite the high efficiency of polymeric nanofibers towards removing dyes and other contaminants, some of these nanofibers have some limitation, such as low mechanical properties, especially when used in harsh environments. Therefore, the incorporation of nanofillers into polymeric nanofibers is an appropriate way to improve their mechanical properties and hydrophilicity. Carbon nanomaterials such as carbon nanotubes, carbon nanofibers and graphene oxide are the most common nanofillers that were used to improve the mechanical properties as well as the adsorption capacity. Sundaran et al. fabricated PU/GO nanofibers using electrospinning and used them for the adsorption of MB and RB dye [169]. It was found the Young’s modulus and tensile strength values of PU/GO nanofibers increased from 0.02 to 0.109 N/mm^2^ and from 2.74 to 11.94 N/mm^2^ with incorporation of 10.0% of GO, respectively. This was attributed to strong interaction between GO sheets and PU chains. Additionally, hydrophilicity was enhanced with increasing GO ratio and became PU nanofibers with a super-hydrophilic nature with 10wt% of GO. The maximum adsorption capacity of PU/GO was 109.8 and 77.15 for removal of MB and RB, respectively. Guo et al. incorporated oxidized MWCNTs into electrospun polyhydroxybutyrate-calcium alginate using electrospinning followed by ionic cross-linking process using CaCl_2_ and was used for the adsorption of Brilliant blue dye [170]. They found that the mechanical and hydrophilic properties were enhanced after incorporation of oxidized MWCNTs and the maximum adsorption capacity also increased from 10.89 to 24.09 mg/g. Ma et al. prepared electrospun PVDF nanofibers and deposited GO onto its surface by ultrasonication and investigated the adsorption of MB [171]. The result showed that the maximum adsorption capacity of PVDF/GO nanofibers was 621.1 mg/g for MB removal with exhibition of good regeneration ability and adsorption stability. Mercante et al. fabricated PMMA-rGO nanofibers by solution blow spinning followed by plasma treatment; and studied its ability towards removal of MB [172]. After plasma treatment, the surface of PMMA nanofibers changed from hydrophobic nature (114°) to hydrophilic nature (41°). The showing maximum adsorption capacity of was 698.51 mg/g for MB. Zhana et al. fabricated PEN/PDA nanofibers and coated GO and used as effective membrane for filtration and separation anionic dye (Direct Blue 14 dye) as shown in Figure 8c [173]. They found that the prepared membrane exhibited good permeate flux, high rejection, antifouling properties and reusability. Despite the contribution of carbon nanomaterials and clays to improving the mechanical and stability properties of EPNFs, their effect is insignificant towards improving their efficiency compared to crosslinked and functionalized EPNFs. However, only graphene oxide was found to play an influential role in improving the adsorption capacity of EPNFs towards removing dyes such as incorporating GO and r-GO into PVDF [171] and PMMA [172], respectively. Table 6 summarize the adsorption data of dyes by EPNFs/carbon nanomaterials and EPNFs/clay nanocomposites.

#### 6.5.3. EPNFs/Silica Nanocomposites

Silica is characterized by large surface area and surface functionalized hydroxyl groups that and is a good nanofillers that was used for enhancing the properties of nanofibers such as surface area, mechanical properties and adsorption property. Li et al. prepared PVA-SH/SiO_2_ nanofiber composites as adsorbents for the removal of indigo carmine and acid red [180]. They found that the adsorption capacity was 246.88 and 81.72 mg/g for indigo carmine and acid red, respectively. Teng et al. fabricated mesoporous PVA/SiO*2*/CD with surface area of 497 m2/g using electrospinning as an adsorbent for the removal of indigo carmine dye [181]. They found that the maximum adsorption capacity was 495 mg/g and the equilibrium contact time was less than 40 min.

#### 6.5.4. EPNFs/Metal Oxides Nanocomposites

Metal oxides are one of the materials that have been used as an adsorbent for removing various pollutants. However, the application of metal oxide NPs for the adsorption of dyes especially in acidic media is still limited due to the dissolution and chemical corrosion at low pH. The incorporation of metal oxides into polymeric nanofibers can solve the corrosion problem of metals oxides as well as the low mechanical properties of polymer nanofibers. Fard et al. incorporated α-Fe_2_O_3_ nanoparticles into poly(vinyl acetate) (PVAc) nanofibers with surface area of 124.5 m^2^/g using electrospinning and in-situ polymerization process as effective adsorbent for the removal of basic red dyes [182]. They presented that the adsorption capacity was 940.57, 946.28 and 912.53 mg/g for the removal of BB41, BB46 and BB18, respectively. Phan et al. fabricated PAN/hinokitiol/ZnO nanofibers using electrospinning and investigated its antibacterial activity and their ability to remove dyes [183]. The maximum adsorption capacity of PAN/hinokitiol/ZnO was 245.76 mg/g and 267.37 mg/g for RR 195 and RB 19, respectively. Xu et al. fabricated PAN/Ag_3_VO_4_/TiO_2_ nanofiber composites as an adsorbent for MB removal. The optimum conditions of MB adsorption onto the surface prepared nanofiber composites were dose of 1 g/L, pH 8, contact time of 20 min and temperature of 25 °C [184]. Additionally, they found that adsorption capacity of PAN/Ag_3_VO_4_/TiO_2_ nanofiber composite was 155.4 mg/g for the removal of MB from an aqueous system.

#### 6.5.5. EPNFs/MOFs Nanocomposites

Metal organic frameworks (MOFs) are a promising class of hybrid material that is constructed from organic linkers and metal–oxide units. MOFs were characterized by high porosity, high surface area, thermal stability, chemical stability and good mechanical properties [185,186]. MOFs are used in many applications such as hydrogen storage [187], catalysis [188,189,190] and water treatment [191,192]. Recently, there is an attention towards the incorporation of MOFs into polymeric materials for the application in water treatment [193,194,195,196]. Li et al. successfully fabricated bio-MOF-1/PAN nanofibers for selective adsorption of methylene blue as cationic dye model as display in Figure 8a [174]. They found that the incorporation of bio-MOF-1 into PAN nanofibers led to enhancement of its mechanical and hydrophilicity properties. The bio-MOF-1/PAN nanofibers exhibited better selectivity for the adsorption and separation towards of MB from mixed dyes aqueous solutions. The significant selectivity in the adsorption and separation was attributed to synergistic effect between nucleophilicity of −C≡N in PAN and anionic charge of bio-MOF-1. Mahmoodi et al. developed novel ZIF-8@PVA/CS nanofibers prepared by electrospinning for the removal of MG dye from aqueous solutions [95]. The maximum adsorption capacity of ZIF-8@PVA/CS nanofibers was 1000 mg/g for MG removal. Zhan et al. fabricated ZIF-8/PAN nanofibers as an effective adsorbent to remove MB and MG dyes from aqueous solutions [92]. They found that the adsorption capacity of ZIF-8/PAN nanofibers towards the removal of MG was ~14 times higher than that of MB removal. This was attributed to the p-p stacking interaction between aromatic ring of MG and two double bonds and a pair of electrons of imidazole moieties in ZIF-8. Jin et al. fabricated ZIF-67/PAN nanofibers with 54% of ZIF-67 by electrospinning and investigated its ability towards the removal of MG, CR and BF dye [197]. They found that the maximum adsorption capacity of ZIF-67/PAN nanofibers was 1305, 849 and 730 mg/g for MG, CR and BF, respectively. Additionally, the results confirmed that the ZIF-67/PAN nanofibers can be used more than four times for the removal of MG. Table 7 displays the adsorption data of dyes by EPNFs/silica [198], EPNFs/metal oxides [199,200,201,202,203,204] and EPNFs/MOFs nanocomposites [92,95,197]. Despite the role of MOFs in improving the ability of some EPNFs to remove some dyes from aqueous solutions, there are some challenges facing the incorporation of MOFs into EPNFs matrix. One of these challenges is low interface bonding between polymers and MOFs due to complex composition of MOFs, low dispersion in solvents of polymers and their different chemical properties with polymers, which makes it difficult to electrospun some polymers directly.

#### 6.5.6. EPNFs/Microorganisms Composite for Dyes Removal

Recently, many studies have been focused on incorporating microorganisms (e.g., bacteria, fungi, algae) into polymeric nanofibers as adsorbents for various pollutants [205,206,207,208]. Despite the ability of free some microorganisms to remove various pollutants, incorporating them into electrospun nanofibers increases their surface area and provides them the possibility of reusing as well as easier handling with nanofibers. However, the number of studies that focused on using of EPNFs/microorganisms nanocomposites to remove dyes from aqueous solutions is still limited. San et al. incorporated three types of bacteria (e.g., *Pseudomonas aeruginosa*, *Aeromonas eucrenophila* and *Clavibacter michiganensis*) into cellulose acetate nanofibers by electrospinning for the removal of MB from wastewater [99]. They found that the efficiency of dye decolorization was 95% after 24 h and was declined to 45% after recycling for four times. In similar study, Keskin et al. developed novel microalgae/polysulfone nanofibers fabricated through electrospinning for the removal of Remazol Black 5 dye [209]. The removal efficiency of microalgae/polysulfone nanofibers was 72.97% and was higher than pristine polysulfone nanofibers (12.36%). Sarioglu et al. encapsulated bacteria (Pseudomonas aeruginosa) into PVA and PEO nanofibers as adsorbents for the remediation of MB dye as displayed in Figure 8b [175]. They found that removal efficiency of bacteria/PEO nanofibers was higher than of bacteria/PVA. Zamel et al. incorporated bacteria (Bacillus paramycoides) into CA/PEO nanofibers by electrospinning technique and used for the decolorization of MB dye [210]. The removal efficiency of bacteria/CA/PEO nanofibers was 93% at first cycle and was decreased to 44% after the 4th cycle. Keskin et al. fabricated and encapsulated living bacteria into cyclodextrin nanofibers for remediation of nickel, chromium and RB-5 dye [211]. They found that the viability of bacteria inside cyclodextrin nanofibers was seven days at 4 °C and the removal efficiency was 82% for decolorization of RB5 dye. Table 8 display removal of efficiency of EPNFs/bacteria nanocomposites for removal organic dyes. Incorporation of bacteria into EPNFs is an early field and needs further study in order to enhance efficiency and reduce the required time to breakdown organic dyes and others pollutants. As we can see in Table 8, more than 24 hours are required for the adsorption and breakdown of dyes by various bacteria/EPNFs.

## 7. EPNFs-Based Carbon Nanofibers as Adsorbents for Dyes Removal

**Figure 9:**
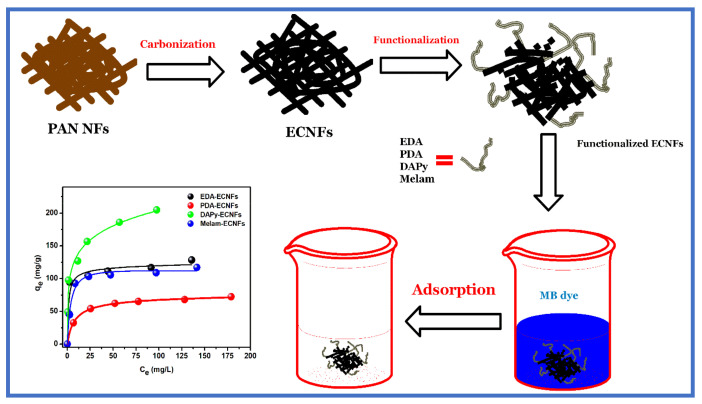
Fabrication and functionalization of ECNFs as a nanoadsorbent for removal of MB dye [217], Copyright © (2020) Elsevier.

Carbon nanofibers (CNFs) have been prepared in the form of mats (webs) from EPNFs by carbonization process and can be used in water treatment. However, the efficiency of pristine CNFs towards removal of ionic pollutants from aqueous solutions is low due to their hydrophobic nature and low dispersion in some solvents as well as their relatively low surface area. Therefore, surface modification is an important approach for enhancing some properties of pristine CNFs and making them more effective towards removal various pollutants. The surface modification of CNMs can be done by different methods, including activation (e.g., physical or chemical) and functionalization processes by various agents. Recently, Thamer et al. reported the fabrication and functionalization of electrospun carbon nanofibers (ECNFs) as novel nanomaterials for the applications in in water treatment including heavy metal removal [213] and dyes removal [214,215,216]. They found that the adsorption capacity of all modified ECNFs for the removal of lead ions, MB, CR and CBB dye was higher than pristine ECNFs. For example, Figure 9 shows the fabrication and functionalization of ECNFs by ethylene diamine (EDA), phenylene diamine (PDA), diamino pyridine (DAPy) and melamine (Melam) and used as adsorbent for removal of MB dye from contaminated water. It was found that the adsorption capacity of DAPy-ECNFs was higher than pristine and others functionalized ECNFs [217]. Recently, oxidized ECNFs has been incorporated into the hydrogel to improve some of its properties such as reducing swelling capacity, improving its mechanical properties, and increasing its efficiency in removing dyes from polluted water [218]. Therefore, ECNFs are promising nanomaterials in the field of water treatment which can be modified their surface properties according to need.

## 8. Conclusions and Future Perspectives

The unique properties of EPNFs such as their high surface area-to-volume ratio, and high porosity nominate them for various applications such as environmental applications as water treatment. Moreover, the introduction of functional groups on its surface can enhance their efficiency towards the removal of pollutants from water especially for the removal of cationic and anionic dyes. In this review, we introduced the applications of electrospun polymers nanofibers and their modified ones as nanoadsorbents for the removal of dyes from aqueous solution. The modified EPNFs by blending, crosslinking, functionalization and incorporation processes showed higher efficiency towards the removal of various dyes and stability in aqueous media than the unmodified one due to the increase in the adsorption sites and enhancing mechanical properties. The review is limited for the in vitro reported studies during the last two decades for using EPNFs as new adsorbents for the removal of dyes for aqueous systems. Moreover, the review discussed the most important factors affecting the adsorption capacity of EPNFs in removing dyes, the adsorption mechanism and different modifying methods for enhancing their stability in aqueous media and the mechanical properties as well as their efficiency. On the other hand, the main challenge for the applications of these materials is their use in filed application via treatment of real wastewater, which contains different types of pollutants. So, the future perspective for using these types of materials can be developed in different ways. The first is the in vitro study for their selectivity in removing dyes. The second is the field applications via their use in treatment of real industrial wastewater. The third is to overcome the low production of electrospun polymers nanofibers in the lab scale, especially with adding inorganic materials as nano-fillers. The fourth, application of nonlinear three-parameters isotherm models to describe the adsorption process as well as conducting column study and not being limited to batch adsorption study. Additionally, another consideration that should be taken for future research is the development of modification methods with the aim of enhancing the surface properties of EPNFs (e.g., surface area, porosity, active functional groups), mechanical properties and their stability in aqueous solutions.

## Figures and Tables

**Table 1:** Optimization of electrospinning conditions for fabricating common polymer nanofibers.

Polymer	Avg. Mw (g/mol)	Concn (*w*/*w*%)	Solvent	Optimum Conditions for Fabricating Bead-Free Nanofibers	Avg. Diameter (nm)	Ref
PVA	130k	7	H_2_O	V = 25 kv, TCD = 5 cm, F. R = 0.1 mL/h	510	[44]
PAN	100k	10	DMF	V = 25 kv, TCD = 20 cm, F. R = 1 mL/h	88	[45]
PVAc	140k	15	EtOH	V = 15 kv, TCD = 10 cm, F. R = 0.06 mL/h	700	[46]
PVP	360k	13	DMF	V = 15 kv, TCD = 20 cm, F. R = 0.25 mL/h	172	[47]
PCL	80k	10	DCM/DMF 3:1	V = 12 kv, TCD = 10 cm, F. R = 1 mL/h	455	[48]
Chitosan	294k	7	AcOH	V = 17 kv, TCD = 16 cm, F. R = 1.6 mL/h	250	[49]
Nylon 6	−	20	Formic acid	V = 15 kv, TCD = 8 cm, F. R = 0.2 mL/h	800	[50]
Poly (St-*co*-AN)	2460k	25	n-Butanone	V = 12 kv, TCD = 23 cm, F. R = 0.2 mL/h	880	[51]
PMMA	120k	15	DMF	V = 12 kv, TCD = 11.4cm, F. R = 2.36 mL/h	177	[52]
CA/PVA	120k	50/50	AcOH	V = 22.5 kv, TCD = 15 cm, F. R = 1.99 mL/h	11	[53]
PA6	17k	20	Formic acid	V = 19 kv, TCD = 10 cm, F. R = 0.9 mL/h	141	[54]

**Table 2:** Effect on adsorption of different dyes on the surface of electrospun polymer nanofibers (EPNFs).

Adsorbent	Dye Class	Dye Name	Temperature Range (K)	Process Type	Ref.
P(NIPAM-co-βCD)/P(NIAPM-co-MAA)	Cationic	MB	298–328	Endothermic	[101]
P(NIPAM-co-MAA)/β-CD	Cationic	CV	298–333	Endothermic	[104]
DCA/PDA	Cationic	MB	288–323	Endothermic	[89]
PMETAC/PES	Anionic	CR	298–318	Endothermic	[83]
PAN-EDA	Anionic	CR	303–323	Endothermic	[67]
EDTA-EDA-PAN	Anionic	MO	298–318	Endothermic	[105]
	Anionic	RR		Exothermic	
sodium alginate	Cationic	MB	288–218	Exothermic	[100]
PES/PEI	Anionic	SY FCF	278–323	Endothermic	[90]
Keratin	Cationic	MB	293–323	Exothermic	[102]
gelatin/β-CD	Cationic	MB	298–333	Exothermic	[103]
PVA/CS/DETA/EDA	Anionic	DR-23	298–333	Endothermic	[106]
PVA/CA/SiO_2_	Anionic	DR-80	298–333	Endothermic	[107]

**Table 3:** Pristine and blend electrospun polymer nanofibers as adsorbent for removal of dyes.

Adsorbent	Dye	Adsorption Conditions				Qmax(mg/g)	Kinetic Model	Isotherm Model	Ref
		pH	T (°C)	Dosage (g/L)	Range conc (mg/L)				
PMAA-*co*-PMMA	MV	6	25	−	5–200	135.37	PSO	L	[119]
Zein	RB5	2–6	25	8	20–200	18.18	PSO	L	[118]
Chitosan	AB-113	–	–	0.66	50–250	1377	PSO	L	[87]
Nylone-6	AB-117	5.5	25	4	25–400	58.8	PSO	F	[113]
polyamide 6	AB-41	−	20	0.1	10	43.9	–	L	[115]
PLLA	MB	–	–	3.33	4–200	8.73	PSO	L	[79]
Keratin	MB	6	20	1	50–250	170	PSO	L	[102]
SPES	MB	6.8	RT	1	6	6.6	PSO	L	[117]
*p*TSA-@PANIPLLA	MO	6	25	1	50–600	377	PFO	L	[114]
CS@PAN	AB-113	–	25	–	50–250	1708	PSO	L	[73]
CS/PA	RB5	1	25	0.2	0–150	456.9	PSO	L	[126]
	P4R					502.4	ELV		
P(β-CD)/PCL	MB	–	RT	0.1	–	10.5	PSO	–	[86]
P(MMA-AA)/PES	MB	9	–	0.25	100–3000 μmol	2257.8	PSO	L	[62]
PANI@N-6	MO	1	RT	–	–	370	–	–	[74]
PDA@CA	MB	6.5	25	0.5	30–100	88.2	PSO	L	[89]
HA@PAN	CV	7	25	0.025	1–7.5 μmol	81.6	–	L	[121]
CS/PA	RB5	1	25	0.2	0–150	198.6	–	L–F	[125]
	P4R					222.4			
P(MMA-co-SSNa)@PES	MB	3–10	RT	0.2	100–500 μmol	625	PSO	L	[128]
PPI-N6	AR-252	4	25	0.6	12.5–100	158.73	PSO	L	[97]
PPy@PVDF/PDA	MB	13	–	–	30–200	370.4	PSO	L	[58]
	CR	1				384.6			
m-PEI/PVDF	MO	7	25	0.5	200–1000	633.3	PSO	L	[127]
DETA@PAN	DR-80	2.1	–	0.044	20–100	1250	PSO	L	[122]
PAN/PAMAM	MO	–	30	3.33	–	120.77	PSO	L	[124]
PAN/PVDF	BB-41	6	25	0.66	10–40	166.6	PSO	L	[85]
CA-PANI/β-CD	MB	8	25	0.64	50–70	49.51	PSO	L	[123]

**Table 4:** Crosslinked EPNFs as adsorbent for removal of dyes.

Adsorbent	Crosslinked Type	Dye	Adsorption Conditions				Qmax (mg/g)	Kinetic Model	Isotherm Model	Ref
			pH	T (°C)	Dos. (g/L)	Range conc (mg/L)				
Na-Alg	CaCl_2_	MB	6	25	0.4	200–1500	2230	PSO	L	[100]
P(NIPAM-co-β-CD)/P(NIAPM-co-MAA)	thermally	MB	9	55	0.35	50–1400	1834.9	PSO	L	[101]
P(HPβCD)/PBA-a	thermally	MB	−	−	1	10–100	46.08	PSO	L	[91]
P(NIPAM-co-MAA)/β-CD	thermally	CV	9	55	0.35	50-900	1253.7	PSO	L	[104]
PVA/CS/DETA/EDA	GA	DR-23	2.1	25	0.1	40–100	526.31	PSO	L	[106]
Pu/PVA/PAA	thermally	MB	11	25	1.33	−	383	PSO	L	[94]
β-CD/PVP	GA	MO	7	25	2.5	10–150	39.82	−	L	[152]
Gel/β-CD	GA	MB	8	25	1.25	5–100	47.4	PSO	L	[103]
Gel/Ca-Alg	CaCl_2_	MB	6	25	0.4	50–900	1937	PSO	L	[144]
PES/PEI	GA	SY FCF	1	30	0.8	100-2000	1000	PSO	L	[90]
		FG FCF					344.83			
		AM					454.55			
PVA–TETA	GA	DR-80	2.1	25	0.06	20–50	128.2	PSO	L	[145]
		DR-81					178.6			
		RR-180					181.8			
Alg/PEO	CaCl_2_	AR-14	1	25	4	−	17.9	−	L	[151]
		BB-41	9				17.3			
PSSNa/PAA@PES	MBA	MB	11		−	50–250 μmol	119.65	PSO	F	[84]
PMETAC@PES	MBA	CR	3	25	−	50-800μmol	208	−	L	[83]
SS/PVA	GA	MB	7	−	−	40–450	223.21	PSO	L	[146]
PEI/EPI/PAN	thermally	MO	−	30	-	-	636.94	−	L	[142]
PVA-CS	GA	DR-80	2.1	25	0.06	20-80	151	PSO	L	[153]
		DR-81					95			
		RR-180					114			
PVA-CS	GA	CR	6	25	6	−	358	PSO	L	[140]
β-CD/PAA/citric acid	thermally	MB	9	20	0.175	80–800	826.45	PSO	L	[147]
PVA-ST	thermally	MB	8.5	25	0.083	25–400	400	PSO	L	[141]
SS/β-CD/PVA	thermally	MB	8	20	0.175	20–200	187.97	PSO	L	[148]
PVA-CS	GA	MO	−	−	5	200–1000	183	−	L	[149]
CA/P(DMDAAC-AM)	MBA	AB-172		25	0.1	20-120	192	PSO	L	[150]
PDA/PEI@PVA/PEI	GA	P-s	7	25	0.5	50–1200	1180	PSO	L	[143]
		MB					1290			

**Table 5:** Functionalized electrospun polymer nanofibers as adsorbent for removal of dyes.

Adsorbent	Dye	Adsorption Conditions				Q_max_ (mg/g)	Kinetic Model	Isotherm Model	Ref	
		pH	T (°C)	Dosage (g/L)	Range Conc (mg/L)					
EDA-g-PAN	MB	−	25	−	−	94.07	PSO	L	[81]	
	ST					110.62				
	RB					138.69				
OX-g-PAN	MB	−	25	−	−	102.15	PSO	L	[82]	
	ST					118.34				
	RB					221.24				
PAN-g-HPEI	MB	10	25	1.66	−	161	PSO	L	[93]	
	MO	5				194				
Carboxylated poly(AN-co-St)	BV-14	6.2	25	2	0–100	67.11	PSO	L	[154]	
PAN-COOH	MG	5	35	0.5	100–500	1038	PSO	L	[157]	
PCD-f-PBST	MB	−	30	1.25	5–100	90.9	PSO	L	[163]	
EDTA-PAN	MO	7	25	2	10–300	90.15	PSO	F	[105]	
						110				
	RB									
CM-β-CD-g-PBSST	MB	9	RT	−	5–200	543.48	PSO	L	[88]	
PAMAM-g-PAN-DETA	DR-80	3.5	RT	0.02	40–100	3333	PSO	L	[72]	
	DR-23					2500				
PHMG-OCS-PVA	CR	−	30	1	−	289	PSO	F	[161]	
AOPAN	MO	3	30	−	10–100	68.07	PFO	L	[155]	
TETA-PPAN	DB-78	2.1	25	0.06	80–140	2500	PSO	L	[158]	
TETA-PAN	DR-80	2.1	RT	0.012	40–100	5000	PSO	L	[156]	
						5000				
	DR-23									
EDA-PAN	CR	3	30	0.5	10–70	130	PSO	L	[67]	
DETA-PAN/PVDF	DR-23	2	−	0.044	20–50	685.63	IPD	L	[159]	
PIM-1	MB	−	−	0.25	50–500	157	−	L	[164]	
TM-PAN	DR-80	3.5	RT	0.033	40–100	1250	PSO	L	[96]	
	DR23					1111				
PDA@PCL/PEO	MB	−	25	0.3	−	14.8	PSO	−	[75]	
	MO					60.2				
PAN/PAMAM	DR80	2.1	25	0.033	40–100	1666.6	PSO	L	[160]	
	DR23					2000				

**Table 6:** EPNFs/clay nanocomposites and EPNFs/carbon nanomaterials as adsorbents for removal of dyes.

Adsorbent	Dye	Adsorption Conditions				Q_max_ (mg/g)	Kinetic Model	Isotherm Model	Ref
		ph	T (°C)	Dosage (g/L)	Range Conc (mg/L)				
PMMA/zeo	MO	−	−	10	30–100	95.33	PSO	L	[167]
CS/PVA/Zeo	MO	4	−	−	100–500	153	PSO	F	[168]
PU/GO	MB	12	30	−	−	109.88	PSO	L	[169]
	RB	10				77.15			
PHB CaAlg/CMWCNT	Bb	−	25	1	5–50	24.09	PSO	F	[170]
PVDF/GO	MB	−	30	0.1	30–200	621.1	PSO	F	[171]
PMMA-rGO	MB	−	RT25	0.3	−	698.51	PSO	L	[172]
PVA/PAA/GO-COOH@PDA	MB	−	25	0.3	10	34.05	PSO	−	[176]
PVA/Gr	CV	−	25	−	1–10	10.96	PSO	L	[177]
PAN/MWCNT-OH	MB	10		1	10–30	8	PSO	F	[178]
P(St-co-AN)/CNTs	MB	8	−	−	5–60	23.55	PSO	L	[179]

**Table 7:** EPNFs/silica, EPNFs/metal oxides and EPNFs/metal organic frameworks (MOFs) nanocomposites as adsorbent for the removal of dyes.

Adsorbent	Dye	Adsorption Conditions				Qmax (mg/g)	Kinetic Model	Isotherm Model	Ref
		pH	T (°C)	Dosage (g/L)	Range Conc (mg/L)				
PVA-SH/SiO_2_	IC	2	25	1	10–500	246.88	PSO	R-P	[180]
	AR-1					81.72	PFO		
PAN-Ti/Ag	MB	8	25	1	5–210	155.4	PSO	L	[184]
APAN/Fe_3_O_4_@3-MPA	IC	5	23	1	5–100	154.5	PFO	L	[199]
PVA/CS/SiO_2_	DR-80	2	RT	0.06	15–30	322	PSO	L	[107]
ZIF-67/PAN	MG	−	RT	0.5	100–600	1305	PSO	F	[197]
SiO_2_@PVA-CD	IC	5.2	RT	1	90–720	495	PSO	L	[181]
PAN/PEI-Fe	CR	−	RT	1	20–60	77.51	PSO	L	[200]
ZIF-8/PAN	MB	11	30	0.25	15–100	120.48	PSO	L	[92]
	MG	5			15–700	1666.6			
PVAc-TEOS@α-Fe_2_O_3_	BR-46	8.5	−	0.035	20–60	946.28	PSO	L	[182]
PVDF@CoAl-LDH	MO	7	30	0.4	20–500	621.17	PSO	L	[201]
PAMAM/α-Fe_2_O_3_	DR-80	3	−	0.032	40–70	1428.5	PSO	L	[202]
	AR-18					1250			
PAN-MoS_2_	RhB	−	−	3	20–1000	75.41	PSO	L	[98]
PAA/SiO_2_	MG	−	30	1	5–300	220.49	PSO	R-P	[198]
PLA@TiO2@MTS	MB	−	RT	−	10–40	236.25	−	−	[203]
CA/CS/SWCNT/Fe_3_O_4_/TiO_2_	MB	3	−	0.5	−	97.6	PSO	L	[204]
	CR					74.2			
ZIF-8@CS/PVA	MG	7	25	0.03	10–40	1000	PSO	L	[95]
ZnO-HT-PAN	RB-19	−	25	0.66	10–400	267.37	PFO	L	[183]
	RR-195					245.76			

**Table 8:** Removal of dyes by EPNFs/bacteria nanocomposites.

Polymer Nanofibers	Microorganisms	Dye	Dye Concentration (ppm)	Removal Efficiency (%)	Time	Ref
Polysulfone	Lysinibacillus sp.	RB-5	30	99.7	24 h	[208]
CA	Aeromonas eucrenophila,	MB	20	95	24 h	[99]
polysulfone	microalgae	RB-5	10	72.97	14 day	[209]
		RB-221	10	30.2		
PVA	Pseudomonas aeruginosa	MB	25	68	48 h	[175]
PEO			25	69		
CA/PEO	Bacillus paramycoides	MB	20	87.39	48 h	[210]
CD	Lysinibacillus sp. NOSK	RB-5	30	82	24 h	[211]
PCL	Clavibacter michiganensis	STB G	200	93.18	48 h	[212]
PLA			200	93.6		

## Data Availability

Not applicable.

## Supplementary Materials

The following are available online at https://www.mdpi.com/2073-4360/13/1/20/s1, Table S1: Chemical structure of dyes included in this review.

## Abbreviations

**Nomenclature**	**Abbreviation**
Acetic acid	AcOH
Acid blue 113	AB-113
Acid blue 117	AB-117
Acid blue 41	AB-41
Acid red 252	AR-252
Alternating current	AC
Amaranth	AM
Amidoximated polyacrilonitrile	APAN
Amidoxime-modified polyacrylonitrile	AOPAN
Azobisisobutyronitrile	AIBN
Basic blue 41	BB-41
Basic fuschin	BF
Basic violet 14	BV-14
Benzoxazine	P(BA-a)
Beta-cyclodextrin	β-CD
Brilliant blue	Bb
Calcium alginate	Ca-Alg
Carboxymethyl-b-cyclodextrin	CM-β-CD
Chitosan	CA
Chitosan	CS
Chitosan/Poly (vinyl alcohol)	CA/PVA
Congo red	CR
Crystal violet	CV
Deacetylated cellulose acetate	DCA
Dichloromethane	DCM
Diethylenetriamine	DETA
Diethylenetriamine	DETA
Dimethyl formamide	DMF
Direct current	DC
Direct red 23	DR-23
Direct red 80	DR-80
Electrospun polymer nanofibers	EPNFs
Epichlorohydrin	EPI
Ethanol	EtOH
Ethylene diamine	EDA
Ethylenediaminetetraacetic	EDTA
Fast green fcf	FG FCF
Flow rate	F. R
Fourier-transform infrared spectroscopy	FTIR
Freundlich model	F
Gelatin	Gel
Glutaraldehyde	GA
Graphene	Gr
Graphene oxide	GO
Hinokitiol	HT
Humic acid	HA
Hyperbranched polyethylenimine	HPEI
Indigo carmine	IC
Intraparticle diffusion	IPD
Langmuir model	L
Layered double hydroxide	LDH
Malachite green	MG
Maximum adsorption capacity	Qmax
Mercaptopropionic acid	MPA
Metal-organic-frameworks	MOFs
Methyl orange	MO
Methylene blue	MB
Methyltrichlorosilane	MTS
Molybdenum disulfide	MoS2
Nanoparticles	NPs
Nylone 6	N-6
Oxidized chitosan	OCS
Oxidized multiwall carbon nanotubes	MWCNTs-COOH
Oxime	OX
Poly ((butylene succinate-co-terephthalate)-co-serinolTerephthalate)	PBSST
Poly ([2- (methacryloyloxy)-ethyl] trimethyl ammonium chloride)	PMETAC
Poly (lactic-co-glycolic acid)	PLGA
Poly (l-lactic acid)	PLLA
Poly (N-Isopropyl acrylamide-co-Methacrylic acid)	P(NIAPM-co-MAA)
Poly (N-Isopropyl acrylamide-co-β-cyclodextrin)	P(NIPAM-co-βCD)
Poly (styrene-co-acrylonitrile)	Poly(St-co-AN)
Poly (vinyl alcohol)	PVA
Poly(2-(dimethylamino)ethyl methacrylate)	PDMAEMA
Poly(amidoamine)	PAMAM
Poly(arylene ether nitrile)(PEN)	PAEN
Poly(butylene succinate-co-terephthalate)	PBST
Poly(hexamethylene guanidine)	PHMG
Poly(hydroxypropyl-β-cyclodextrin)	P(HPβCD)
Poly(methacrylic acid)	PMAA
Poly(methyl methacrylate-co-acrylic acid)	P(MMA-co-AA)
Poly(propylene imine)	PPI
Poly(γ-benzyl-L-glutamate)	PBGL
Polyacrylamide	PAM
Polyacrylonitrile	PAN
Polyamide	PA
Polyamide 6	PA6
Polyaniline	PANI
Polycaprolactone	PCL
Polydopamine	PDA
Polyether sulfone	PES
Polyethylene oxide	PEO
Polyethylene terephthalate	PET
Polyethyleneimine	PEI
Polymer of intrinsic microporosity	PIM-1
Polymethyl methacrylate	PMMA
Polypyrrole	PPy
Polystyrene	PS
Polyurethane	PU
Polyvinyl acetate	PVAc
Polyvinyl chloride	PVC
Polyvinyl pyrrolidone	PVP
Polyvinylidene fluoride	PVDF
Ponceau 4R	P4R
Ponceau s	P-s
Pseudo first order model	PFO
Pseudo second order model	PSO
P-toluenesulfonic acid	pTSA
Pullulan	Pu
Reactive black 5	RB-5
Reactive blue 180	RR-180
Reactive red	RR
Redlich-Peterson model	R-P
Reduced graphene oxide	rGO
Rhodamine B	RhB
Safranin T	ST
Sericin	SS
Sodium alginate	Na-Alg
Sodium styrene sulfonate	SSNa
Sulfonated polysulfone	SPES
Sunset yellow fcf	SY FCF
Tectomer	TM
Tetraethyl orthosilicate	TEOS
Thiol-functionalized polyvinyl alcohol	PVA-SH
Tip-collector-distance	TCD
Titanium dioxide	TiO2
Triethylenetetramine	TETA
Volatage	V
X-ray photoelectron spectroscopy	XPS
Zeolite	Zeo
Zeolitic imidazolate frameworks	ZIF
