# Supplementary material for: Modified Electrospun Polymeric Nanofibers and Their Nanocomposites as Nanoadsorbents for Toxic Dye Removal from Contaminated Waters: A Review
Source: Polymers (Basel). 2020 Dec 23;13(1):20. doi: 10.3390/polym13010020 (PMC7793529; doi:10.3390/polym13010020)
Supplement: Supplementary file 1 [file polymers-13-00020-s001.pdf]

Review

Modified Electrospun Polymeric nanofibers and their nanocomposites as nanoadsorbents for toxic dyes removal from contaminated waters: A review

Badr M. Thamer, Ali Aldalbahi*, Meera Moydeen A, Mostafizur Rahaman and Mohamed H. El-Newehy

Department of Chemistry, College of Science, King Saud University, Riyadh 11451, Saudi Arabia

bthamer@ksu.edu.sa (B.M.T.); aaldalbahi@ksu.edu.sa (A.A.); malhameed@ksu.edu.sa (M.MA.); mrahaman@ksu.edu.sa (M.R.); melnewehy@ksu.edu.sa (M.H.E.)

***Corresponding author; E-mail: aaldalbahi@ksu.edu.sa (A.A.)**

Received: 03 November 2020; Accepted: date; Published: date

Table S1: Chemical structure of dyes included in this review.

Name	Class	MW (g/mol)	λ_{max} (nm)	Structure
Acid blue 113 (AB-113)	Anionic	681.65	560	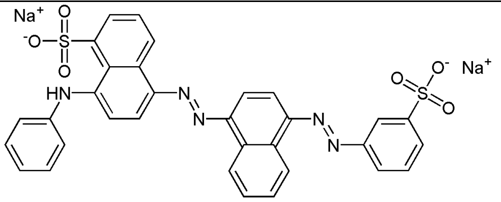
Acid blue 117 (AB-117)	Anionic	594.57	588	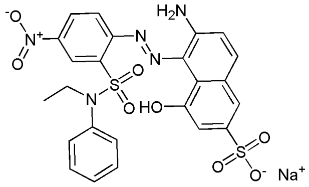
Acid black 172 (AB-172)	Anionic	993.71	572	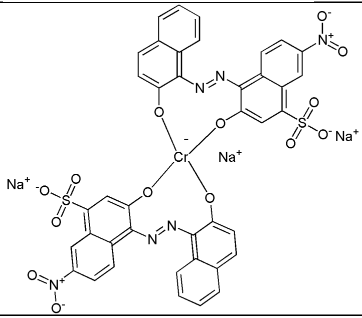

Name	Class	MW (g/mol)	λ_{max} (nm)	Structure
Acid blue 41 (AB-41)	Anionic	487.5		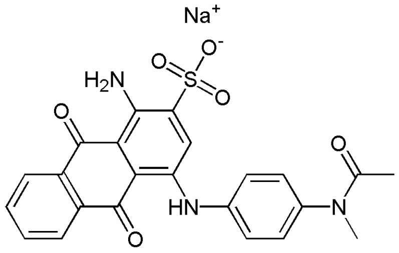
Amaranth (Am)	Anionic	604.473	521	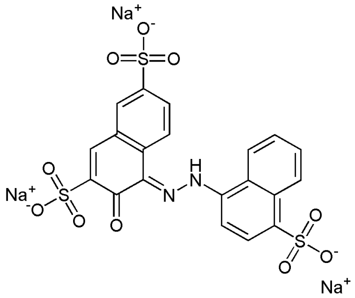
Acid red 1 (AR-1)	Anionic	509.42	531	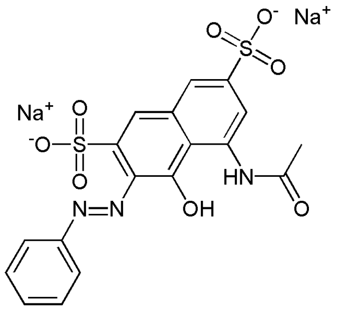
Acid red 18 (AR-18)	Anionic	604.479	506	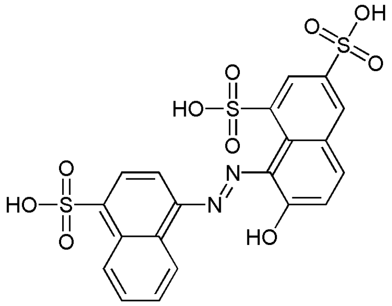
Basic Blue 41 (BB-41)	Anionic	482.57	606	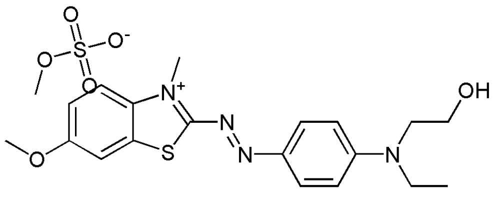

Name	Class	MW (g/mol)	λ_{max} (nm)	Structure
Basic Red 46 (BR-46)	Cationic	401.3	530	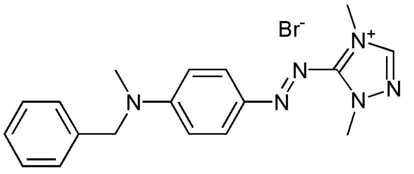
Basic violet 14 (BV-14)	Cationic	337.8	545	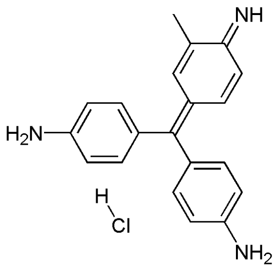
Congo red (CR)	Anionic	696.66	498	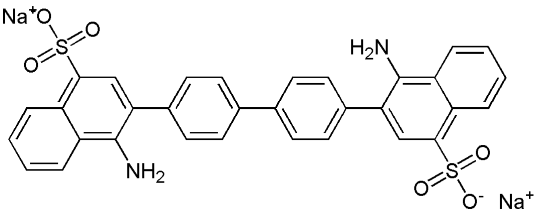
Crystal violet (CV)	Cationic	407.97	590	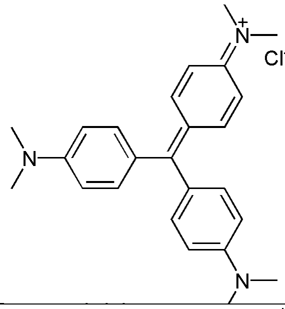
Direct Blue 78 (DB-78)	Anionic	1055.9	604	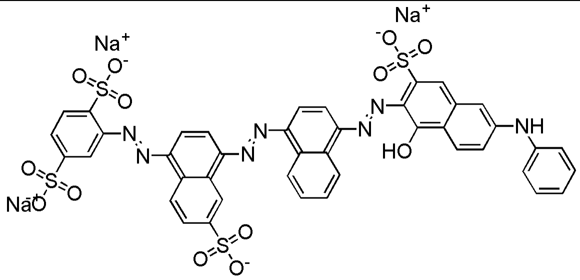

Name	Class	MW (g/mol)	λ_{max} (nm)	Structure
Direct red 23 (DR-23)	Anionic	813.72	507	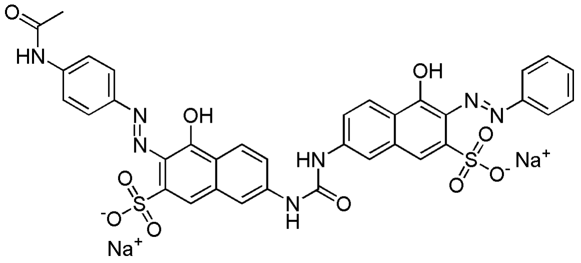
Direct red 80 (DR-80)	Anionic	1373	528	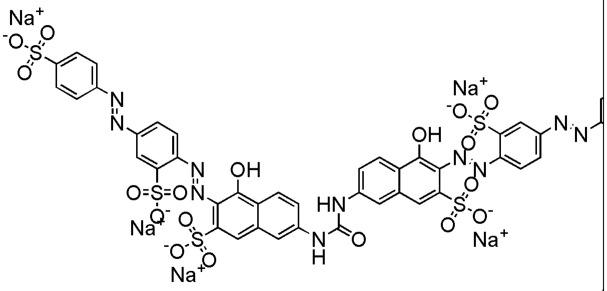
Fast Green FCF (FG-FCF))	Anionic	808.85	624	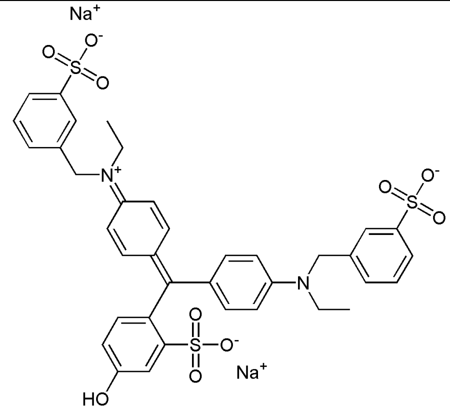
Indigo carmine (IC)	Anionic	466.37	611	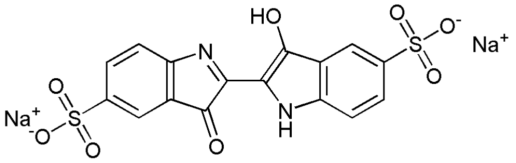
Methylene blue (MB)	Cationic	319.85	664	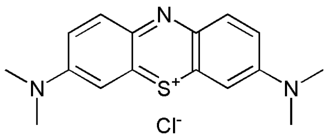

**Name**	**Class**	**MW (g/mol)**	**λ_{max} (nm)**	**Structure**
Malachite Green (MG)	Cationic	364.9	617	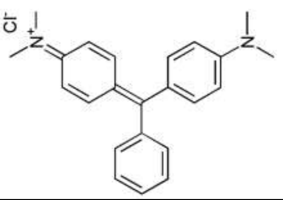
Methyl orange (MO)	Anionic	327.3	465	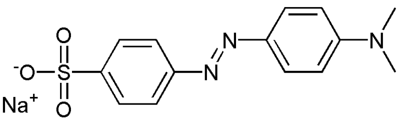
Methyl violet (MV)	Cationic	393.95	583	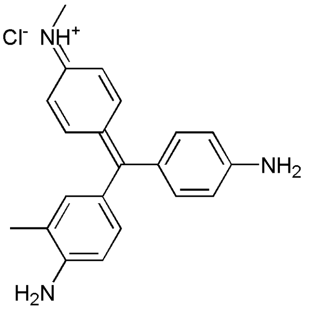
Ponceau 4R P-4P	Anionic	604.47	505	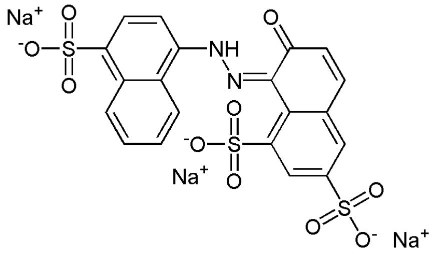
Ponceau s (P-s)	Anionic	672.63	523	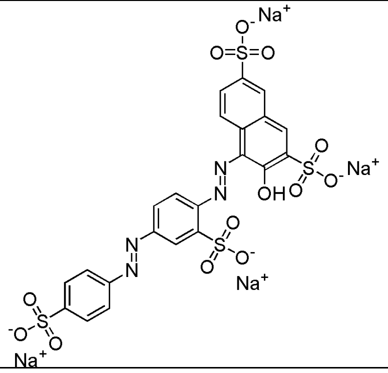

Name	Class	MW (g/mol)	λ_{max} (nm)	Structure
Reactive blue 19 (RB-19)	Anionic	626.54	596	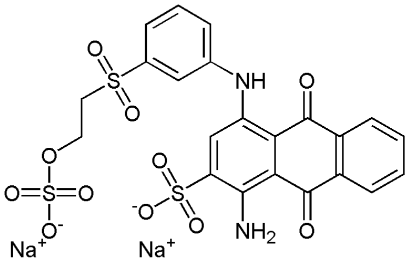
Reactive blue 195 (RB-195)	Anionic	1021.4	541	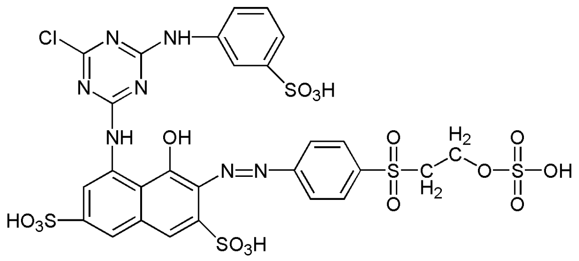
Reactive blue 221 (RB-221)	Anionic	890	601	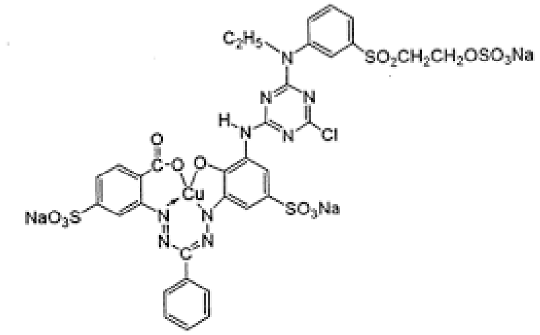
Reactive black 5 (RB-5)	Anionic	991.8	597	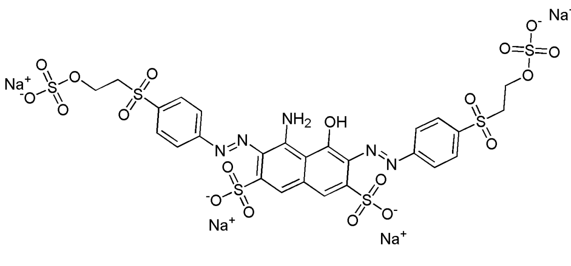
Rhodmine B (RhB)	Cationic	479.02	554	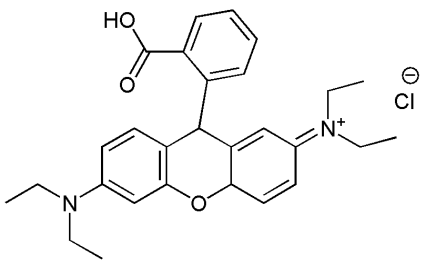

Name	Class	MW (g/mol)	λ_{max} (nm)	Structure
Reactive Red 180 (RR-180)	Anionic	933.76	542	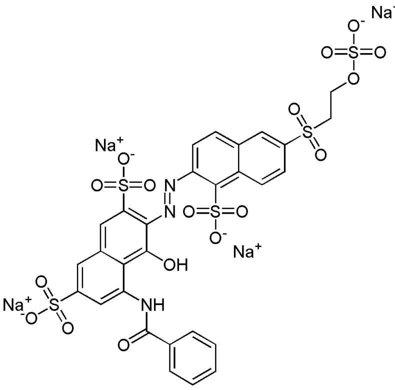
Reactive Red 195 (RR-195)	Anionic	1136.3	517	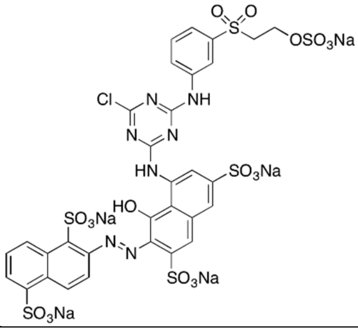
Safranin T (S-T)	Cationic	350.84	520	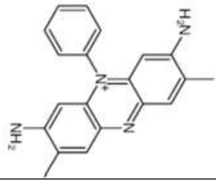
Sunset Yellow FCF (SY-FCF)	Anionic	452.38	482	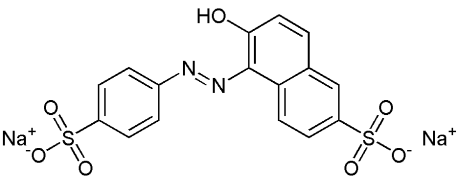
Setazol Turquoise Blue G (STB-G)	Anionic	1289	626	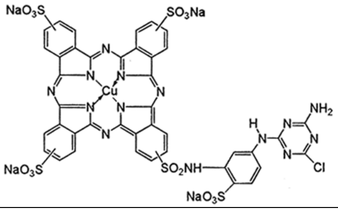
